# Supplementary material for: Association of fluid balance with mortality in sepsis is modified by admission hemoglobin levels: A large database study
Source: PLoS One. 2021 Jun 14;16(6):e0252629. doi: 10.1371/journal.pone.0252629 (PMC8202933; doi:10.1371/journal.pone.0252629)
Supplement: S1 Fig — (a) Distribution of Hb measurements. (b) Time of Hb measurement after ICU admission (Hours). (c) Number of Hb measurements per patient in the first 24 hours after ICU admission. (d) Distribution of Hb change (%) in the first 24 hours after ICU admission. Hb change (%) was calculated as (admission Hb- last Hb in 24 hours)/admission Hb, where Hb measures after blood transfusion were not taken into consideration. Abbrieviations: Hb = hemoglobin. (DOCX) [file pone.0252629.s001.docx]

**S1 Fig. Distribution of hemoglobin measurements.** (a) Distribution of Hb measurements. (b) Time of Hb measurement after ICU admission (Hours). (c) Number of Hb measurements per patient in the first 24 hours after ICU admission. (d) Distribution of Hb change (%) in the first 24 hours after ICU admission. Hb change (%) was calculated as (*admission Hb- last Hb in 24 hours)/admission Hb*, where Hb measures after blood transfusion were not taken into consideration. Abbreviations: Hb = hemoglobin.
