# Supplementary material for: Association of fluid balance with mortality in sepsis is modified by admission hemoglobin levels: A large database study
Source: PLoS One. 2021 Jun 14;16(6):e0252629. doi: 10.1371/journal.pone.0252629 (PMC8202933; doi:10.1371/journal.pone.0252629)
Supplement: S2 Fig — Logistic regression results for subgroups. The ORs represent the risk of 28-day mortality for sepsis patients at different observation windows after ICU admission. Subgroups from a to h are as follows: moderate anemia patients with congestive heart failure, patients without moderate anemia with congestive heart failure, moderate anemia patients with mechanical ventilation at admission, patients without moderate anemia with mechanical ventilation at admission, moderate anemia patients without mechanical ventilation at admission, patients without moderate anemia without mechanical ventilation at admission, moderate anemia patients with chronic kidney disease, patients without moderate anemia with chronic kidney disease. For moderate anemia patients with congestive heart failure, an increased risk of 28-day mortality was observed at 24 hours (OR 1.09, 95% CI 1.09–3.42, p = 0.03) with increasing fluid balance (median 1.47L, IQR 0.76L–3.32L). For patients without moderate anemia and with congestive heart failure, there was no significant difference in risk of 28-day mortality (OR 1.01, CI 0.95–1.06, p = 0.737 at 24 hours) with increasing fluid balance. For moderate anemia patients with chronic kidney disease, there was no significant difference in risk of 28-day mortality (OR 1.09, 95% CI 0.98–1.21, p = 0.124 at 24 hours) with increasing fluid balance. For patients without moderate anemia and with chronic kidney disease, there was no significant difference in risk of 28-day morality (OR 1.06, 95% CI 0.97–1.16, p = 0.201 at 24 hours) with increasing fluid balance. For moderate anemia patients and without mechanical ventilation at ICU admission, an increased risk of 28-day mortality was observed at 24 hours (OR 1.08, 95% CI 1.03–1.13, p = 0.003) with increasing fluid balance (median 1.72L IQR 0.76L – 3.27L). For patients without moderate anemia and without mechanical ventilation on admission, there was a decreased risk of 28-day mortality (OR 0.9, 95% CI 0.83–0.97, p = 0.0 [file pone.0252629.s002.docx]

**S2 Fig. Visualization of regression results for subgroups of sepsis patients at different observation windows after ICU admission.** Logistic regression results for subgroups. The ORs represent the risk of 28-day mortality for sepsis patients at different observation windows after ICU admission. Subgroups from a to h are as follows: moderate anemia patients with congestive heart failure, patients without moderate anemia with congestive heart failure, moderate anemia patients with mechanical ventilation at admission, patients without moderate anemia with mechanical ventilation at admission, moderate anemia patients without mechanical ventilation at admission, patients without moderate anemia without mechanical ventilation at admission, moderate anemia patients with chronic kidney disease, patients without moderate anemia with chronic kidney disease. For moderate anemia patients with congestive heart failure, an increased risk of 28-day mortality was observed at 24 hours (OR 1.09, 95% CI 1.09 - 3.42, p = 0.03) with increasing fluid balance (median 1.47L, IQR 0.76L - 3.32L). For patients without moderate anemia and with congestive heart failure, there was no significant difference in risk of 28-day mortality (OR 1.01, CI 0.95 - 1.06, p=0.737 at 24 hours) with increasing fluid balance. For moderate anemia patients with chronic kidney disease, there was no significant difference in risk of 28-day mortality (OR 1.09, 95% CI 0.98 – 1.21, p=0.124 at 24 hours) with increasing fluid balance. For patients without moderate anemia and with chronic kidney disease, there was no significant difference in risk of 28-day morality (OR 1.06, 95% CI 0.97 – 1.16, p=0.201 at 24 hours) with increasing fluid balance. For moderate anemia patients and without mechanical ventilation at ICU admission, an increased risk of 28-day mortality was observed at 24 hours (OR 1.08, 95% CI 1.03 - 1.13, p=0.003) with increasing fluid balance (median 1.72L IQR 0.76L – 3.27L). For patients without moderate anemia and without mechanical ventilation on admission, there was a decreased risk of 28-day mortality (OR 0.9, 95% CI 0.83 - 0.97, p=0.009 at 6 hours; OR 0.94, 95% CI 0.89 – 0.98, p = 0.011 at 12 hours) with increasing fluid balance (median 1.15L, IQR 0.49L – 2.25L at 12 hours). For moderate anemia patients with mechanical ventilation on ICU admission, there was no significant difference in risk of 28-day mortality (OR 0.96, 95% CI 0.85 – 1.07, p = 0.45 at 24 hours) with increasing fluid balance. For patients without moderate anemia and with mechanical ventilation on ICU admission, there was no significant difference in risk of 28-day mortality (OR 0.97, 95% CI 0.9 – 1.04, p = 0.429 at 42 hours) with increasing fluid balance. Abbreviations: CHF = Congestive heart failure; CKD = Chronic kidney disease; FB = Fluid balance; ICU = Intensive Care Unit; MV = Mechanical ventilation; OR = Odds Ratio;
