# Supplementary material for: Association of fluid balance with mortality in sepsis is modified by admission hemoglobin levels: A large database study
Source: PLoS One. 2021 Jun 14;16(6):e0252629. doi: 10.1371/journal.pone.0252629 (PMC8202933; doi:10.1371/journal.pone.0252629)
Supplement: S3 Fig — Logistic regression results for sepsis patients without blood transfusion. The ORs represent the risk of 28-day mortality for sepsis patients who did not receive blood transfusion in their ICU stay, at different observation windows after ICU admission. (a) and (b) show the regression results for patients who did not undergo blood transfusion, with and without moderate anemia, respectively. Magnitude of risk of 28-day mortality was higher in patients with moderate anemia (OR 1.17, 95% CI 1.04–1.31, p = 0.009 at 24 hours) compared to patients without moderate anemia (OR 1.05, 95% CI 1–1.1, p = 0.046 at 24 hours) with increasing fluid balance. For moderate anemia patients with congestive heart failure, there was increased risk of 28-day mortality at 24 hours (OR 1.28, 95% CI 1.02–1.62, p = 0.036). For patients without moderate anemia with congestive heart failure, there was no significant difference in risk of 28-day mortality with increasing fluid balance. For moderate anemia patients without mechanical ventilation on ICU admission, there was increased risk of 28-day mortality at 18 hours (OR 1.17, 95% CI 1.01–1.36, p = 0.038) and 24 hours (OR 1.21, 95% CI 1.06–1.37, p = 0.004) with an increasing fluid balance. For patients without moderate anemia without mechanical ventilation on ICU admission, there was no significant difference in risk of 28-day mortality with an increasing fluid balance. Abbreviations: FB = Fluid balance; ICU = Intensive Care Unit; OR = Odds Ratio. (DOCX) [file pone.0252629.s003.docx]

1. **Moderate anemia patients b. Patients without moderate anemia**

**S3 Fig. Visualization of sensitivity analyses results for patients without blood transfusion in their ICU stay.** Logistic regression results for sepsis patients without blood transfusion. The ORs represent the risk of 28-day mortality for sepsis patients who did not receive blood transfusion in their ICU stay, at different observation windows after ICU admission. (a) and (b) show the regression results for patients who did not undergo blood transfusion, with and without moderate anemia, respectively. Magnitude of risk of 28-day mortality was higher in patients with moderate anemia (OR 1.17, 95% CI 1.04 – 1.31, p=0.009 at 24 hours) compared to patients without moderate anemia (OR 1.05, 95% CI 1 – 1.1, p=0.046 at 24 hours) with increasing fluid balance. For moderate anemia patients with congestive heart failure, there was increased risk of 28-day mortality at 24 hours (OR 1.28, 95% CI 1.02 – 1.62, p=0.036). For patients without moderate anemia with congestive heart failure, there was no significant difference in risk of 28-day mortality with increasing fluid balance. For moderate anemia patients without mechanical ventilation on ICU admission, there was increased risk of 28-day mortality at 18 hours (OR 1.17, 95% CI 1.01 – 1.36, p=0.038) and 24 hours (OR 1.21, 95% CI 1.06 – 1.37, p = 0.004) with an increasing fluid balance. For patients without moderate anemia without mechanical ventilation on ICU admission, there was no significant difference in risk of 28-day mortality with an increasing fluid balance. Abbreviations: FB = Fluid balance; ICU = Intensive Care Unit; OR = Odds Ratio
