# Supplementary material for: Association of fluid balance with mortality in sepsis is modified by admission hemoglobin levels: A large database study
Source: PLoS One. 2021 Jun 14;16(6):e0252629. doi: 10.1371/journal.pone.0252629 (PMC8202933; doi:10.1371/journal.pone.0252629)
Supplement: S4 Fig — Logistic regression results for extending to 48 hours of ICU admission. The ORs represent the risk of 28-day mortality for sepsis patients at different observation windows after ICU admission. (a) and (b) show the regression results for patients with and without moderate anemia respectively. There was an increased risk of 28-day mortality at 24 hours of ICU admission and beyond (OR 1.05, 95% CI 1.01–1.1, p = 0.027 at 24 hours; OR 1.08, 95% CI 1.04–1.11, p<0.001 at 48 hours) with increasing fluid balance in patients with moderate anemia. In patients without moderate anemia, there was a decreased risk of 28-day mortality (OR 0.87, 95% CI 0.81–0.94, p<0.001 at 6 hours; OR 0.92, 95% CI 0.87–0.96, p<0.001 at 12 hours; OR 0.95, 95% CI 0.92–0.99, p = 0.013 at 18 hours) with increasing fluid balance. Abbreviations: FB = Fluid balance; ICU = Intensive Care Unit; OR = Odds Ratio. (DOCX) [file pone.0252629.s004.docx]

1. **Moderate anemia patients b. Patients without moderate anemia**

**S4 Fig. Visualization of sensitivity analyses results extending to 48 hours of ICU admission.** Logistic regression results for extending to 48 hours of ICU admission. The ORs represent the risk of 28-day mortality for sepsis patients at different observation windows after ICU admission. (a) and (b) show the regression results for patients with and without moderate anemia respectively. There was an increased risk of 28-day mortality at 24 hours of ICU admission and beyond (OR 1.05, 95% CI 1.01 – 1.1, p=0.027 at 24 hours; OR 1.08, 95% CI 1.04 – 1.11, p<0.001 at 48 hours) with increasing fluid balance in patients with moderate anemia. In patients without moderate anemia, there was a decreased risk of 28-day mortality (OR 0.87, 95% CI 0.81 – 0.94, p<0.001 at 6 hours; OR 0.92, 95% CI 0.87 – 0.96, p<0.001 at 12 hours; OR 0.95, 95% CI 0.92 – 0.99, p=0.013 at 18 hours) with increasing fluid balance. Abbreviations: FB = Fluid balance; ICU = Intensive Care Unit; OR = Odds Ratio
