# Supplementary material for: Association of fluid balance with mortality in sepsis is modified by admission hemoglobin levels: A large database study
Source: PLoS One. 2021 Jun 14;16(6):e0252629. doi: 10.1371/journal.pone.0252629 (PMC8202933; doi:10.1371/journal.pone.0252629)
Supplement: S5 Fig — Comparison of Odds Ratio of logistic regressions applying four imputation methods. The ORs represent the risk of 28-day mortality for sepsis patients at different observation windows after ICU admission. (a) and (b) show the regression results for patients with and without moderate anemia using four imputation methods including K-nearest neighbors, mean, mode and median imputation. Abbreviations: OR = FB = Fluid balance; Odds Ratio; KNN = K-nearest neighbors. (DOCX) [file pone.0252629.s005.docx]

1. **Moderate anemia patients b. Patients without moderate anemia**

**S5 Fig. Sensitivity analyses results for imputation methods.** Comparison of Odds Ratio of logistic regressions applying four imputation methods. The ORs represent the risk of 28-day mortality for sepsis patients at different observation windows after ICU admission. (a) and (b) show the regression results for patients with and without moderate anemia using four imputation methods including K-nearest neighbors, mean, mode and median imputation. Abbreviations: OR = FB = Fluid balance; Odds Ratio; KNN = K-nearest neighbors
