# Supplementary material for: Association of fluid balance with mortality in sepsis is modified by admission hemoglobin levels: A large database study
Source: PLoS One. 2021 Jun 14;16(6):e0252629. doi: 10.1371/journal.pone.0252629 (PMC8202933; doi:10.1371/journal.pone.0252629)
Supplement: S1 Table — (DOCX) [file pone.0252629.s006.docx]

**S1 Table. Regression results for subgroups of sepsis patients at different observation windows after ICU admission**

| Subgroup | Observation window | Patient Number | OR (95% CI) | Median fluid balance, L (Median [IQR]) | Median hemoglobin, g/dL (Median [IQR]) |
| --- | --- | --- | --- | --- | --- |
| Congestive heart failure patients | **6 hours** | 1962 | 1.01 (0.9, 1.12) p = 0.897 | 0.77 (0.34, 1.45) | 10.3 (8.9, 11.6) |
|  | **12 hours** | 1962 | 0.99 (0.93, 1.07) p = 0.86 | 1.13 (0.51, 2.27) | 10.3 (9.3, 11.38) |
|  | **18 hours** | 1955 | 1.02 (0.97, 1.08) p = 0.463 | 1.42 (0.7, 2.94) | 10.3 (9.2, 11.4) |
|  | **24 hours** | 1902 | 1.04 (0.99, 1.09) p = 0.119 | 1.69 (0.8, 3.4) | 9.95 (9.1, 11.17) |
| Moderate anemia patients with Congestive heart failure | **6 hours** | 672 | 1.06 (0.9, 1.26) p = 0.468 | 0.75 (0.3, 1.42) | 8.75 (7.9, 9.6) |
|  | **12 hours** | 672 | 1.04 (0.92, 1.17) p = 0.512 | 0.98 (0.44, 2.35) | 8.9 (8.3, 9.5) |
|  | **18 hours** | 671 | 1.07 (0.97, 1.18) p = 0.164 | 1.3 (0.66, 2.94) | 8.9 (8, 9.5) |
|  | **24 hours** | 643 | 1.09 (1.01, 1.18) p = 0.03 | 1.47 (0.76, 3.32) | 9 (8.4, 9.5) |
| Without moderate anemia, with Congestive heart failure | **6 hours** | 1290 | 0.98 (0.85, 1.13) p = 0.78 | 0.79 (0.36, 1.46) | 11 (10, 12) |
|  | **12 hours** | 1290 | 0.97 (0.88, 1.06) p = 0.481 | 1.19 (0.55, 2.25) | 10.8 (9.9, 11.8) |
|  | **18 hours** | 1284 | 1 (0.93, 1.07) p = 0.893 | 1.48 (0.72, 2.95) | 10.65 (9.9, 11.72) |
|  | **24 hours** | 1259 | 1.01 (0.95, 1.07) p = 0.737 | 1.76 (0.85, 3.43) | 10.4 (9.4, 11.53) |
| All patients with mechanical ventilation | **6 hours** | 1150 | 0.89 (0.78, 1) p = 0.055 | 0.94 (0.42, 1.93) | 11 (9.7, 12.3) |
|  | **12 hours** | 1150 | 0.93 (0.85, 1.01) p = 0.095 | 1.45 (0.54, 2.82) | 10.4 (9.25, 11.95) |
|  | **18 hours** | 1149 | 0.95 (0.89, 1.02) p = 0.189 | 1.96 (0.91, 3.57) | 10.35 (9.2, 11.62) |
|  | **24 hours** | 1145 | 0.96 (0.91, 1.02) p = 0.233 | 2.29 (1.02, 4.18) | 10.65 (9.33, 11.97) |
| Moderate anemia patients with mechanical ventilation | **6 hours** | 314 | 0.84 (0.66, 1.06) p = 0.153 | 1.01 (0.42, 1.78) | 9.2 (8.6, 9.8) |
|  | **12 hours** | 314 | 0.92 (0.78, 1.07) p = 0.289 | 1.36 (0.47, 2.74) | 8.9 (8.35, 9.65) |
|  | **18 hours** | 313 | 0.94 (0.82, 1.07) p = 0.351 | 2.01 (1, 3.78) | 9.1 (8.4, 9.7) |
|  | **24 hours** | 311 | 0.96 (0.85, 1.07) p = 0.45 | 2.6 (1.06, 4.37) | 8.5 (8.15, 8.8) |
| Without moderate anemia, with mechanical ventilation | **6 hours** | 836 | 0.91 (0.78, 1.06) p = 0.224 | 0.87 (0.42, 1.93) | 11.5 (10.5, 12.8) |
|  | **12 hours** | 836 | 0.95 (0.85, 1.05) p = 0.289 | 1.47 (0.59, 2.85) | 11.05 (9.9, 12.4) |
|  | **18 hours** | 836 | 0.97 (0.89, 1.05) p = 0.44 | 1.93 (0.9, 3.48) | 10.8 (9.65, 11.9) |
|  | **24 hours** | 834 | 0.97 (0.9, 1.04) p = 0.429 | 2.2 (1.02, 4.11) | 11 (9.75, 12.1) |
| All patients without mechanical ventilation | **6 hours** | 6982 | 0.93 (0.88, 1) p = 0.036 | 0.77 (0.36, 1.43) | 10.2 (8.8, 11.6) |
|  | **12 hours** | 6976 | 0.96 (0.92, 1) p = 0.066 | 1.15 (0.5, 2.25) | 10.3 (9.2, 11.6) |
|  | **18 hours** | 6954 | 1 (0.96, 1.03) p = 0.825 | 1.44 (0.66, 2.89) | 10.2 (9.15, 11.4) |
|  | **24 hours** | 6738 | 1.02 (0.99, 1.04) p = 0.283 | 1.75 (0.77, 3.38) | 10.1 (9, 11.3) |
| Moderate anemia patients without mechanical ventilation | **6 hours** | 2155 | 1.01 (0.9, 1.13) p = 0.865 | 0.79 (0.35, 1.43) | 8.7 (7.9, 9.6) |
|  | **12 hours** | 2152 | 1.02 (0.95, 1.1) p = 0.563 | 1.12 (0.5, 2.26) | 8.7 (8, 9.4) |
|  | **18 hours** | 2146 | 1.06 (1, 1.12) p = 0.053 | 1.4 (0.64, 2.8) | 8.8 (8, 9.7) |
|  | **24 hours** | 2064 | 1.08 (1.03, 1.13) p = 0.003 | 1.72 (0.76, 3.27) | 8.8 (8.1, 9.5) |
| Without moderate anemia, without mechanical ventilation | **6 hours** | 4827 | 0.9 (0.83, 0.97) p = 0.009 | 0.76 (0.36, 1.42) | 10.8 (9.6, 12.1) |
|  | **12 hours** | 4824 | 0.94 (0.89, 0.98) p = 0.011 | 1.15 (0.49, 2.25) | 10.9 (9.9, 11.9) |
|  | **18 hours** | 4808 | 0.97 (0.93, 1.01) p = 0.129 | 1.47 (0.67, 2.93) | 10.6 (9.7, 11.8) |
|  | **24 hours** | 4674 | 0.99 (0.96, 1.02) p = 0.515 | 1.78 (0.78, 3.43) | 10.5 (9.5, 11.7) |
| All patients with CKD | **6 hours** | 1337 | 0.88 (0.74, 1.03) p = 0.119 | 0.7 (0.31, 1.24) | 9.8 (8.5, 10.97) |
|  | **12 hours** | 1335 | 0.99 (0.89, 1.09) p = 0.789 | 1 (0.43, 2) | 9.8 (8.8, 11.07) |
|  | **18 hours** | 1329 | 1.05 (0.96, 1.13) p = 0.274 | 1.27 (0.56, 2.54) | 9.9 (8.85, 11.05) |
|  | **24 hours** | 1283 | 1.07 (1, 1.14) p = 0.054 | 1.52 (0.67, 2.94) | 9.6 (8.9, 10.7) |
| Moderate anemia patients with CKD | **6 hours** | 536 | 0.89 (0.68, 1.14) p = 0.374 | 0.71 (0.3, 1.28) | 8.7 (7.8, 9.4) |
|  | **12 hours** | 535 | 1 (0.85, 1.17) p = 0.976 | 1.01 (0.44, 2.19) | 8.7 (8, 9.2) |
|  | **18 hours** | 532 | 1.05 (0.92, 1.2) p = 0.424 | 1.21 (0.57, 2.65) | 8.6 (8.03, 9.7) |
|  | **24 hours** | 510 | 1.09 (0.98, 1.21) p = 0.124 | 1.4 (0.57, 2.89) | 8.9 (8.3, 9.4) |
| Without moderate anemia, with CKD | **6 hours** | 801 | 0.91 (0.72, 1.12) p = 0.372 | 0.69 (0.33, 1.22) | 10.5 (9.3, 11.5) |
|  | **12 hours** | 800 | 0.99 (0.85, 1.14) p = 0.865 | 0.99 (0.42, 1.95) | 10.6 (9.7, 11.7) |
|  | **18 hours** | 797 | 1.05 (0.94, 1.17) p = 0.383 | 1.31 (0.54, 2.44) | 10.3 (9.3, 11.3) |
|  | **24 hours** | 773 | 1.06 (0.97, 1.16) p = 0.201 | 1.58 (0.74, 2.94) | 10.1 (9.4, 11.2) |
