# Supplementary material for: Association of fluid balance with mortality in sepsis is modified by admission hemoglobin levels: A large database study
Source: PLoS One. 2021 Jun 14;16(6):e0252629. doi: 10.1371/journal.pone.0252629 (PMC8202933; doi:10.1371/journal.pone.0252629)
Supplement: S2 Table — (DOCX) [file pone.0252629.s007.docx]

**S2 Table. Sensitivity analyses results for patients without blood transfusion in their ICU stay**

| Subgroup | Observation window | Patient Number | OR (95% CI) | Median fluid balance, L  (Median [IQR]) | Median hemoglobin, g/dL  (Median [IQR]) |
| --- | --- | --- | --- | --- | --- |
| All patients | **6 hours** | 4183 | 0.95 (0.86, 1.04) p = 0.271 | 0.7 (0.33, 1.24) | 11.2 (10, 12.6) |
|  | **12 hours** | 4179 | 1.01 (0.95, 1.07) p = 0.806 | 0.98 (0.43, 1.88) | 10.9 (9.8, 12.05) |
|  | **18 hours** | 4161 | 1.04 (0.99, 1.1) p = 0.082 | 1.23 (0.54, 2.41) | 10.7 (9.7, 11.9) |
|  | **24 hours** | 4000 | 1.06 (1.02, 1.11) p = 0.006 | 1.42 (0.62, 2.81) | 10.6 (9.5, 11.8) |
| Moderate anemia patients | **6 hours** | 753 | 1.13 (0.87, 1.46) p = 0.355 | 0.65 (0.3, 1.07) | 9.3 (8.7, 9.8) |
|  | **12 hours** | 751 | 1.13 (0.94, 1.34) p = 0.191 | 0.84 (0.38, 1.61) | 9.1 (8.5, 9.6) |
|  | **18 hours** | 747 | 1.13 (0.99, 1.3) p = 0.074 | 1.04 (0.5, 2.1) | 9.3 (8.6, 9.9) |
|  | **24 hours** | 698 | 1.17 (1.04, 1.31) p = 0.009 | 1.32 (0.52, 2.52) | 9.1 (8.28, 9.7) |
| Patients without moderate anemia | **6 hours** | 3430 | 0.93 (0.84, 1.03) p = 0.168 | 0.72 (0.34, 1.28) | 11.6 (10.5, 12.8) |
|  | **12 hours** | 3428 | 0.99 (0.93, 1.06) p = 0.867 | 1.01 (0.45, 1.95) | 11.2 (10.2, 12.3) |
|  | **18 hours** | 3414 | 1.03 (0.98, 1.09) p = 0.222 | 1.27 (0.56, 2.46) | 11 (10, 12.2) |
|  | **24 hours** | 3302 | 1.05 (1, 1.1) p = 0.046 | 1.46 (0.64, 2.85) | 10.9 (9.8, 12.1) |
| Congestive heart failure patients | **6 hours** | 971 | 1.06 (0.87, 1.27) p = 0.563 | 0.7 (0.35, 1.25) | 11.1 (10, 12.2) |
|  | **12 hours** | 971 | 1.08 (0.96, 1.22) p = 0.179 | 1 (0.44, 1.84) | 10.8 (9.9, 11.8) |
|  | **18 hours** | 967 | 1.1 (1, 1.21) p = 0.039 | 1.21 (0.65, 2.35) | 10.6 (9.5, 11.72) |
|  | **24 hours** | 932 | 1.1 (1.02, 1.19) p = 0.013 | 1.34 (0.69, 2.69) | 10.5 (9.4, 11.6) |
| Moderate anemia patients with Congestive heart failure | **6 hours** | 187 | 1.39 (0.83, 2.32) p = 0.203 | 0.65 (0.26, 1.05) | 9.1 (8.6, 9.8) |
|  | **12 hours** | 187 | 1.32 (0.92, 1.91) p = 0.126 | 0.76 (0.32, 1.29) | 9.1 (8.67, 9.72) |
|  | **18 hours** | 187 | 1.28 (0.97, 1.69) p = 0.076 | 0.96 (0.54, 1.85) | 9.1 (8.6, 9.5) |
|  | **24 hours** | 175 | 1.28 (1.02, 1.62) p = 0.036 | 1.1 (0.42, 2.11) | 9.3 (9.03, 9.65) |
| Without moderate anemia, with Congestive heart failure | **6 hours** | 784 | 1.01 (0.81, 1.24) p = 0.954 | 0.72 (0.36, 1.28) | 11.4 (10.6, 12.5) |
|  | **12 hours** | 784 | 1.05 (0.92, 1.19) p = 0.457 | 1.08 (0.49, 1.98) | 10.95 (10.3, 12.07) |
|  | **18 hours** | 780 | 1.08 (0.98, 1.2) p = 0.128 | 1.26 (0.67, 2.52) | 11 (9.9, 11.9) |
|  | **24 hours** | 757 | 1.09 (0.99, 1.18) p = 0.064 | 1.38 (0.74, 2.81) | 10.9 (9.6, 11.8) |
| All patients with mechanical ventilation | **6 hours** | 549 | 0.89 (0.71, 1.12) p = 0.322 | 0.73 (0.35, 1.52) | 11.7 (10.4, 13) |
|  | **12 hours** | 549 | 1.04 (0.88, 1.22) p = 0.652 | 1.15 (0.42, 2.37) | 11.2 (10.1, 12.85) |
|  | **18 hours** | 548 | 1.1 (0.96, 1.25) p = 0.165 | 1.44 (0.56, 2.77) | 11.1 (9.72, 12.17) |
|  | **24 hours** | 545 | 1.09 (0.98, 1.22) p = 0.123 | 1.73 (0.75, 3.08) | 11 (10, 12) |
| Moderate anemia patients with mechanical ventilation | **6 hours** | 87 | 0.7 (0.29, 1.43) p = 0.347 | 0.59 (0.32, 1.06) | 9.5 (8.9, 9.8) |
|  | **12 hours** | 87 | 1.03 (0.6, 1.77) p = 0.925 | 0.76 (0.25, 1.54) | 9.1 (8.55, 9.53) |
|  | **18 hours** | 86 | 1.06 (0.67, 1.7) p = 0.791 | 1.22 (0.59, 1.77) | 9.55 (8.8, 9.93) |
|  | **24 hours** | 85 | 1.09 (0.74, 1.62) p = 0.664 | 1.38 (0.56, 2.23) | 10.2 (9.2, 10.4) |
| Without moderate anemia, with mechanical ventilation | **6 hours** | 462 | 0.93 (0.72, 1.18) p = 0.539 | 0.75 (0.35, 1.62) | 11.9 (10.78, 13.22) |
|  | **12 hours** | 462 | 1.04 (0.88, 1.24) p = 0.638 | 1.23 (0.46, 2.49) | 11.8 (10.55, 13.05) |
|  | **18 hours** | 462 | 1.1 (0.96, 1.27) p = 0.181 | 1.56 (0.57, 2.84) | 11.25 (10.03, 12.47) |
|  | **24 hours** | 460 | 1.09 (0.96, 1.23) p = 0.171 | 1.79 (0.76, 3.12) | 11.05 (10, 12) |
| All patients without mechanical ventilation | **6 hours** | 3634 | 0.95 (0.85, 1.05) p = 0.33 | 0.7 (0.33, 1.21) | 11.1 (9.9, 12.5) |
|  | **12 hours** | 3630 | 1 (0.93, 1.06) p = 0.887 | 0.96 (0.44, 1.82) | 10.8 (9.7, 12) |
|  | **18 hours** | 3613 | 1.03 (0.98, 1.09) p = 0.267 | 1.18 (0.54, 2.38) | 10.7 (9.7, 11.9) |
|  | **24 hours** | 3455 | 1.05 (1, 1.1) p = 0.03 | 1.4 (0.6, 2.77) | 10.5 (9.4, 11.8) |
| Moderate anemia patients without mechanical ventilation | **6 hours** | 666 | 1.28 (0.96, 1.7) p = 0.083 | 0.65 (0.3, 1.06) | 9.3 (8.6, 9.8) |
|  | **12 hours** | 664 | 1.17 (0.96, 1.42) p = 0.109 | 0.86 (0.4, 1.63) | 9.1 (8.5, 9.62) |
|  | **18 hours** | 661 | 1.17 (1.01, 1.36) p = 0.038 | 1 (0.48, 2.12) | 9.3 (8.6, 9.9) |
|  | **24 hours** | 613 | 1.21 (1.06, 1.37) p = 0.004 | 1.31 (0.52, 2.54) | 9.1 (8.3, 9.7) |
| Without moderate anemia, without mechanical ventilation | **6 hours** | 2968 | 0.92 (0.82, 1.03) p = 0.14 | 0.71 (0.34, 1.24) | 11.5 (10.5, 12.8) |
|  | **12 hours** | 2966 | 0.98 (0.91, 1.05) p = 0.531 | 0.98 (0.45, 1.87) | 11.1 (10.2, 12.2) |
|  | **18 hours** | 2952 | 1.01 (0.96, 1.08) p = 0.622 | 1.23 (0.55, 2.42) | 11 (10, 12.2) |
|  | **24 hours** | 2842 | 1.04 (0.98, 1.09) p = 0.176 | 1.42 (0.62, 2.82) | 10.9 (9.8, 12.1) |
| All patients with CKD | **6 hours** | 660 | 0.87 (0.65, 1.14) p = 0.333 | 0.69 (0.29, 1.05) | 10.7 (9.5, 11.9) |
|  | **12 hours** | 660 | 1.01 (0.83, 1.2) p = 0.954 | 0.89 (0.4, 1.59) | 10.4 (9.4, 11.5) |
|  | **18 hours** | 656 | 1.1 (0.96, 1.26) p = 0.159 | 1.1 (0.5, 2.16) | 10.3 (9.7, 11.3) |
|  | **24 hours** | 622 | 1.12 (1, 1.26) p = 0.045 | 1.25 (0.57, 2.61) | 9.85 (9.2, 11.2) |
| Moderate anemia patients with CKD | **6 hours** | 177 | 0.97 (0.45, 1.96) p = 0.936 | 0.54 (0.22, 0.91) | 9.2 (8.8, 9.7) |
|  | **12 hours** | 177 | 1 (0.6, 1.62) p = 0.997 | 0.82 (0.31, 1.31) | 9 (8.7, 9.5) |
|  | **18 hours** | 175 | 1.15 (0.77, 1.69) p = 0.498 | 0.9 (0.41, 1.5) | 8.8 (8.6, 9.7) |
|  | **24 hours** | 160 | 1.2 (0.85, 1.67) p = 0.287 | 0.8 (0.4, 1.84) | 9.15 (8.57, 9.4) |
| Without moderate anemia, with CKD | **6 hours** | 483 | 0.85 (0.6, 1.16) p = 0.323 | 0.71 (0.37, 1.13) | 11.1 (10.2, 12.5) |
|  | **12 hours** | 483 | 0.99 (0.79, 1.22) p = 0.934 | 0.93 (0.44, 1.71) | 10.8 (10, 11.83) |
|  | **18 hours** | 481 | 1.1 (0.93, 1.28) p = 0.25 | 1.2 (0.53, 2.29) | 10.85 (9.9, 11.4) |
|  | **24 hours** | 462 | 1.12 (0.98, 1.27) p = 0.101 | 1.4 (0.68, 2.77) | 10.6 (9.53, 11.67) |
