# Supplementary material for: Association of fluid balance with mortality in sepsis is modified by admission hemoglobin levels: A large database study
Source: PLoS One. 2021 Jun 14;16(6):e0252629. doi: 10.1371/journal.pone.0252629 (PMC8202933; doi:10.1371/journal.pone.0252629)
Supplement: S3 Table — (DOCX) [file pone.0252629.s008.docx]

**S3 Table. Sensitivity analyses results for extending to 48 hours of ICU admission**

| Subgroup | Observation window | Patient Number | OR (95% CI) | Median fluid balance, L (Median [IQR]) | Median hemoglobin, g/dL (Median [IQR]) |
| --- | --- | --- | --- | --- | --- |
| All patients | **6 hours** | 8004 | 0.91 (0.86, 0.96) p = 0.002 | 0.78 (0.36, 1.47) | 10.2 (8.9, 11.7) |
|  | **12 hours** | 7998 | 0.94 (0.91, 0.98) p = 0.004 | 1.17 (0.5, 2.31) | 10.3 (9.2, 11.6) |
|  | **18 hours** | 7975 | 0.98 (0.95, 1.01) p = 0.175 | 1.49 (0.67, 2.97) | 10.2 (9.2, 11.5) |
|  | **24 hours** | 7759 | 1 (0.97, 1.02) p = 0.772 | 1.8 (0.79, 3.44) | 10.2 (9.1, 11.4) |
|  | **30 hours** | 7417 | 1.01 (0.99, 1.03) p = 0.347 | 2.1 (0.92, 3.98) | 10.1 (9, 11.2) |
|  | **36 hours** | 7172 | 1.02 (1, 1.04) p = 0.044 | 2.33 (0.99, 4.4) | 10 (9, 11.1) |
|  | **42 hours** | 6942 | 1.03 (1.01, 1.05) p = 0.005 | 2.53 (1.08, 4.76) | 10.1 (9, 11.3) |
|  | **48 hours** | 6589 | 1.03 (1.02, 1.05) p < 0.001 | 2.76 (1.16, 5.24) | 10 (9, 10.9) |
| Moderate anemia patients | **6 hours** | 2426 | 0.99 (0.89, 1.09) p = 0.827 | 0.8 (0.35, 1.49) | 8.8 (7.9, 9.6) |
|  | **12 hours** | 2423 | 1.01 (0.94, 1.08) p = 0.877 | 1.15 (0.5, 2.32) | 8.7 (8, 9.5) |
|  | **18 hours** | 2416 | 1.04 (0.98, 1.1) p = 0.167 | 1.45 (0.66, 2.91) | 8.9 (8, 9.7) |
|  | **24 hours** | 2333 | 1.05 (1.01, 1.1) p = 0.027 | 1.76 (0.78, 3.4) | 8.7 (8.1, 9.4) |
|  | **30 hours** | 2214 | 1.07 (1.02, 1.11) p = 0.002 | 2.17 (0.96, 3.88) | 8.6 (8, 9.5) |
|  | **36 hours** | 2133 | 1.07 (1.03, 1.11) p < 0.001 | 2.29 (0.97, 4.33) | 8.7 (8, 9.4) |
|  | **42 hours** | 2059 | 1.08 (1.04, 1.12) p < 0.001 | 2.44 (1.13, 4.74) | 8.7 (8.15, 9.6) |
|  | **48 hours** | 1936 | 1.08 (1.04, 1.11) p < 0.001 | 2.63 (1.14, 5.06) | 8.9 (8.05, 9.7) |
| Patients without moderate anemia | **6 hours** | 5578 | 0.87 (0.81, 0.94) p < 0.001 | 0.77 (0.37, 1.47) | 10.9 (9.7, 12.2) |
|  | **12 hours** | 5575 | 0.92 (0.87, 0.96) p < 0.001 | 1.18 (0.5, 2.31) | 10.9 (9.9, 12) |
|  | **18 hours** | 5559 | 0.95 (0.92, 0.99) p = 0.013 | 1.51 (0.67, 2.99) | 10.6 (9.7, 11.8) |
|  | **24 hours** | 5426 | 0.97 (0.94, 1) p = 0.076 | 1.82 (0.8, 3.46) | 10.6 (9.6, 11.8) |
|  | **30 hours** | 5203 | 0.99 (0.96, 1.01) p = 0.364 | 2.08 (0.92, 4.02) | 10.4 (9.5, 11.4) |
|  | **36 hours** | 5039 | 1 (0.98, 1.02) p = 0.99 | 2.35 (1.01, 4.44) | 10.3 (9.38, 11.4) |
|  | **42 hours** | 4883 | 1.01 (0.98, 1.03) p = 0.657 | 2.6 (1.07, 4.76) | 10.4 (9.5, 11.5) |
|  | **48 hours** | 4653 | 1.02 (0.99, 1.04) p = 0.139 | 2.83 (1.17, 5.29) | 10.2 (9.2, 11.1) |
| Congestive heart failure patients | **6 hours** | 1935 | 0.96 (0.86, 1.08) p = 0.496 | 0.76 (0.33, 1.44) | 10.3 (8.9, 11.6) |
|  | **12 hours** | 1935 | 0.96 (0.89, 1.04) p = 0.318 | 1.12 (0.51, 2.25) | 10.3 (9.28, 11.33) |
|  | **18 hours** | 1928 | 1 (0.94, 1.06) p = 0.922 | 1.4 (0.69, 2.91) | 10.2 (9.2, 11.35) |
|  | **24 hours** | 1876 | 1.02 (0.97, 1.07) p = 0.484 | 1.65 (0.8, 3.35) | 9.9 (9.1, 11.1) |
|  | **30 hours** | 1814 | 1.03 (0.98, 1.07) p = 0.244 | 1.9 (0.88, 3.79) | 9.9 (8.8, 10.8) |
|  | **36 hours** | 1773 | 1.03 (0.99, 1.07) p = 0.147 | 2.13 (0.97, 4.27) | 10.1 (9, 11.1) |
|  | **42 hours** | 1726 | 1.03 (0.99, 1.07) p = 0.101 | 2.31 (1.04, 4.53) | 10.1 (9.1, 11) |
|  | **48 hours** | 1649 | 1.04 (1, 1.07) p = 0.032 | 2.59 (1.12, 4.96) | 9.85 (9.07, 10.72) |
| Moderate anemia patients with Congestive heart failure | **6 hours** | 664 | 1.06 (0.89, 1.26) p = 0.498 | 0.75 (0.29, 1.42) | 8.8 (7.9, 9.6) |
|  | **12 hours** | 664 | 1.04 (0.92, 1.17) p = 0.539 | 0.99 (0.45, 2.34) | 8.9 (8.3, 9.5) |
|  | **18 hours** | 663 | 1.07 (0.97, 1.17) p = 0.198 | 1.3 (0.67, 2.94) | 8.9 (8.2, 9.5) |
|  | **24 hours** | 635 | 1.09 (1.01, 1.18) p = 0.035 | 1.45 (0.76, 3.32) | 9 (8.4, 9.5) |
|  | **30 hours** | 607 | 1.1 (1.03, 1.18) p = 0.006 | 1.75 (0.83, 3.69) | 8.8 (8.1, 9.5) |
|  | **36 hours** | 587 | 1.1 (1.03, 1.17) p = 0.005 | 1.87 (0.88, 4.21) | 8.8 (8.3, 9.5) |
|  | **42 hours** | 571 | 1.1 (1.03, 1.16) p = 0.002 | 2.11 (1, 4.54) | 8.7 (8.4, 9.1) |
|  | **48 hours** | 545 | 1.1 (1.04, 1.16) p = 0.001 | 2.23 (1, 4.98) | 8.4 (8.05, 9.75) |
| Without moderate anemia, with Congestive heart failure | **6 hours** | 1271 | 0.9 (0.77, 1.05) p = 0.183 | 0.77 (0.35, 1.45) | 10.9 (10, 12) |
|  | **12 hours** | 1271 | 0.92 (0.83, 1.01) p = 0.081 | 1.16 (0.54, 2.21) | 10.8 (9.9, 11.8) |
|  | **18 hours** | 1265 | 0.96 (0.89, 1.03) p = 0.254 | 1.46 (0.71, 2.9) | 10.6 (9.9, 11.7) |
|  | **24 hours** | 1241 | 0.98 (0.92, 1.04) p = 0.457 | 1.71 (0.83, 3.36) | 10.4 (9.4, 11.53) |
|  | **30 hours** | 1207 | 0.98 (0.93, 1.04) p = 0.536 | 1.95 (0.91, 3.85) | 10.2 (9.2, 11) |
|  | **36 hours** | 1186 | 0.99 (0.94, 1.04) p = 0.74 | 2.25 (1.04, 4.27) | 10.4 (9.5, 11.5) |
|  | **42 hours** | 1155 | 0.99 (0.95, 1.04) p = 0.771 | 2.39 (1.12, 4.48) | 10.3 (9.6, 11.15) |
|  | **48 hours** | 1104 | 1 (0.96, 1.05) p = 0.879 | 2.68 (1.18, 4.94) | 10 (9.2, 10.9) |
| All patients with mechanical ventilation | **6 hours** | 1135 | 0.88 (0.77, 1) p = 0.046 | 0.94 (0.42, 1.91) | 11 (9.7, 12.3) |
|  | **12 hours** | 1135 | 0.93 (0.85, 1.01) p = 0.099 | 1.44 (0.54, 2.79) | 10.4 (9.22, 11.9) |
|  | **18 hours** | 1134 | 0.96 (0.89, 1.02) p = 0.202 | 1.96 (0.91, 3.59) | 10.35 (9.2, 11.6) |
|  | **24 hours** | 1130 | 0.96 (0.9, 1.02) p = 0.199 | 2.27 (1.02, 4.18) | 10.65 (9.38, 12) |
|  | **30 hours** | 1114 | 0.98 (0.93, 1.03) p = 0.355 | 2.53 (1.11, 4.58) | 10 (9.05, 10.95) |
|  | **36 hours** | 1102 | 0.99 (0.94, 1.04) p = 0.631 | 2.75 (1.24, 5.22) | 10.05 (9.1, 11.1) |
|  | **42 hours** | 1092 | 1 (0.96, 1.04) p = 0.972 | 3 (1.36, 5.64) | 9.75 (8.8, 11) |
|  | **48 hours** | 1057 | 1.01 (0.97, 1.05) p = 0.627 | 3.16 (1.45, 6.1) | 10 (9.05, 10.85) |
| Moderate anemia patients with mechanical ventilation | **6 hours** | 310 | 0.84 (0.66, 1.05) p = 0.144 | 1.01 (0.42, 1.92) | 9.15 (8.6, 9.8) |
|  | **12 hours** | 310 | 0.9 (0.76, 1.06) p = 0.222 | 1.36 (0.47, 2.74) | 9 (8.4, 9.67) |
|  | **18 hours** | 309 | 0.93 (0.81, 1.07) p = 0.314 | 2.03 (1, 3.81) | 9.1 (8.4, 9.75) |
|  | **24 hours** | 307 | 0.95 (0.84, 1.07) p = 0.402 | 2.6 (1.04, 4.41) | 8.5 (8.1, 8.8) |
|  | **30 hours** | 301 | 0.97 (0.87, 1.07) p = 0.519 | 3.2 (1.21, 4.95) | 8.8 (7.85, 9.5) |
|  | **36 hours** | 298 | 0.98 (0.89, 1.08) p = 0.739 | 3.03 (1.21, 5.48) | 8.6 (8.05, 9.3) |
|  | **42 hours** | 297 | 1 (0.92, 1.09) p = 0.924 | 3.49 (1.68, 5.83) | 8.5 (8.1, 8.95) |
|  | **48 hours** | 286 | 1.02 (0.94, 1.11) p = 0.572 | 3.42 (1.6, 6.16) | 9 (8.3, 9.82) |
| Without moderate anemia, with mechanical ventilation | **6 hours** | 825 | 0.9 (0.77, 1.05) p = 0.205 | 0.87 (0.41, 1.91) | 11.6 (10.5, 12.78) |
|  | **12 hours** | 825 | 0.95 (0.85, 1.06) p = 0.363 | 1.47 (0.59, 2.85) | 11.05 (9.9, 12.4) |
|  | **18 hours** | 825 | 0.97 (0.89, 1.06) p = 0.524 | 1.93 (0.9, 3.48) | 10.7 (9.65, 11.9) |
|  | **24 hours** | 823 | 0.97 (0.9, 1.04) p = 0.421 | 2.18 (1, 4.1) | 11 (9.7, 12.1) |
|  | **30 hours** | 813 | 0.98 (0.92, 1.05) p = 0.593 | 2.42 (1.08, 4.48) | 10.35 (9.5, 11.2) |
|  | **36 hours** | 804 | 0.99 (0.94, 1.05) p = 0.833 | 2.62 (1.25, 5.1) | 10.5 (9.5, 11.3) |
|  | **42 hours** | 795 | 1 (0.95, 1.05) p = 0.976 | 2.86 (1.18, 5.52) | 10 (9.2, 11.2) |
|  | **48 hours** | 771 | 1.01 (0.96, 1.06) p = 0.671 | 3.03 (1.36, 6.06) | 10.2 (9.1, 11.05) |
| All patients without mechanical ventilation | **6 hours** | 6869 | 0.91 (0.85, 0.98) p = 0.009 | 0.77 (0.36, 1.42) | 10.1 (8.8, 11.6) |
|  | **12 hours** | 6863 | 0.94 (0.9, 0.99) p = 0.01 | 1.15 (0.49, 2.23) | 10.3 (9.2, 11.6) |
|  | **18 hours** | 6841 | 0.98 (0.95, 1.02) p = 0.302 | 1.43 (0.65, 2.86) | 10.2 (9.15, 11.4) |
|  | **24 hours** | 6629 | 1 (0.97, 1.03) p = 0.87 | 1.73 (0.75, 3.36) | 10.1 (9.1, 11.3) |
|  | **30 hours** | 6303 | 1.02 (0.99, 1.04) p = 0.172 | 2.03 (0.88, 3.85) | 10.1 (9, 11.2) |
|  | **36 hours** | 6070 | 1.03 (1, 1.05) p = 0.019 | 2.26 (0.96, 4.27) | 10 (9, 11.1) |
|  | **42 hours** | 5850 | 1.03 (1.01, 1.05) p = 0.003 | 2.42 (1.04, 4.6) | 10.1 (9.1, 11.3) |
|  | **48 hours** | 5532 | 1.04 (1.02, 1.06) p < 0.001 | 2.67 (1.12, 5.04) | 10 (9, 10.9) |
| Moderate anemia patients without mechanical ventilation | **6 hours** | 2116 | 1.03 (0.92, 1.15) p = 0.631 | 0.78 (0.35, 1.42) | 8.8 (7.9, 9.6) |
|  | **12 hours** | 2113 | 1.03 (0.95, 1.11) p = 0.439 | 1.11 (0.5, 2.25) | 8.7 (8, 9.4) |
|  | **18 hours** | 2107 | 1.07 (1, 1.13) p = 0.04 | 1.38 (0.64, 2.79) | 8.8 (8, 9.7) |
|  | **24 hours** | 2026 | 1.08 (1.02, 1.14) p = 0.004 | 1.7 (0.75, 3.24) | 8.8 (8.1, 9.5) |
|  | **30 hours** | 1913 | 1.09 (1.04, 1.14) p < 0.001 | 2.06 (0.91, 3.7) | 8.6 (8, 9.5) |
|  | **36 hours** | 1835 | 1.09 (1.05, 1.14) p < 0.001 | 2.19 (0.95, 4.08) | 8.7 (8, 9.4) |
|  | **42 hours** | 1762 | 1.1 (1.06, 1.14) p < 0.001 | 2.3 (1.04, 4.5) | 8.8 (8.2, 9.6) |
|  | **48 hours** | 1650 | 1.09 (1.05, 1.13) p < 0.001 | 2.47 (1.08, 4.94) | 8.9 (8, 9.7) |
| Without moderate anemia, without mechanical ventilation | **6 hours** | 4753 | 0.86 (0.79, 0.93) p < 0.001 | 0.75 (0.36, 1.41) | 10.8 (9.6, 12.1) |
|  | **12 hours** | 4750 | 0.91 (0.86, 0.96) p < 0.001 | 1.15 (0.49, 2.2) | 10.9 (9.9, 11.9) |
|  | **18 hours** | 4734 | 0.95 (0.91, 0.99) p = 0.01 | 1.45 (0.66, 2.89) | 10.6 (9.7, 11.8) |
|  | **24 hours** | 4603 | 0.97 (0.93, 1) p = 0.088 | 1.74 (0.75, 3.39) | 10.5 (9.6, 11.7) |
|  | **30 hours** | 4390 | 0.99 (0.95, 1.02) p = 0.365 | 2.01 (0.88, 3.97) | 10.4 (9.45, 11.4) |
|  | **36 hours** | 4235 | 1 (0.97, 1.03) p = 0.941 | 2.3 (0.97, 4.33) | 10.3 (9.3, 11.45) |
|  | **42 hours** | 4088 | 1 (0.98, 1.03) p = 0.761 | 2.53 (1.04, 4.67) | 10.4 (9.5, 11.6) |
|  | **48 hours** | 3882 | 1.01 (0.99, 1.04) p = 0.221 | 2.76 (1.15, 5.14) | 10.2 (9.4, 11.12) |
| All patients with CKD | **6 hours** | 1321 | 0.88 (0.74, 1.04) p = 0.147 | 0.7 (0.32, 1.25) | 9.7 (8.5, 10.9) |
|  | **12 hours** | 1319 | 0.99 (0.89, 1.1) p = 0.834 | 1 (0.43, 2) | 9.8 (8.8, 11.1) |
|  | **18 hours** | 1313 | 1.05 (0.96, 1.14) p = 0.281 | 1.27 (0.56, 2.54) | 9.9 (8.8, 11) |
|  | **24 hours** | 1267 | 1.07 (0.99, 1.14) p = 0.074 | 1.52 (0.67, 2.94) | 9.6 (8.9, 10.7) |
|  | **30 hours** | 1194 | 1.07 (1.01, 1.14) p = 0.024 | 1.72 (0.77, 3.48) | 9.4 (8.6, 10.5) |
|  | **36 hours** | 1144 | 1.08 (1.02, 1.14) p = 0.005 | 2.01 (0.87, 3.76) | 9.4 (8.72, 10.6) |
|  | **42 hours** | 1098 | 1.08 (1.03, 1.14) p = 0.002 | 2.15 (0.99, 4.06) | 9.9 (9, 11.22) |
|  | **48 hours** | 1042 | 1.08 (1.03, 1.14) p = 0.001 | 2.38 (1.06, 4.53) | 9.6 (8.67, 10.43) |
| Moderate anemia patients with CKD | **6 hours** | 532 | 0.92 (0.7, 1.18) p = 0.535 | 0.71 (0.3, 1.28) | 8.7 (7.8, 9.4) |
|  | **12 hours** | 531 | 1.02 (0.87, 1.19) p = 0.804 | 1.02 (0.43, 2.2) | 8.7 (8, 9.2) |
|  | **18 hours** | 528 | 1.07 (0.94, 1.22) p = 0.313 | 1.22 (0.58, 2.66) | 8.6 (8.03, 9.7) |
|  | **24 hours** | 506 | 1.1 (0.99, 1.22) p = 0.089 | 1.4 (0.58, 2.91) | 8.9 (8.25, 9.4) |
|  | **30 hours** | 477 | 1.1 (1, 1.21) p = 0.045 | 1.7 (0.71, 3.52) | 8.4 (7.82, 9.3) |
|  | **36 hours** | 455 | 1.11 (1.02, 1.21) p = 0.018 | 1.85 (0.84, 3.91) | 8.75 (8, 9.4) |
|  | **42 hours** | 437 | 1.12 (1.04, 1.22) p = 0.005 | 2.02 (0.97, 4.05) | 8.95 (8.4, 9.9) |
|  | **48 hours** | 414 | 1.12 (1.04, 1.21) p = 0.003 | 2.18 (1, 4.5) | 8.9 (8.2, 9.6) |
| Without moderate anemia, with CKD | **6 hours** | 789 | 0.9 (0.71, 1.11) p = 0.334 | 0.69 (0.34, 1.23) | 10.4 (9.3, 11.5) |
|  | **12 hours** | 788 | 0.97 (0.84, 1.13) p = 0.74 | 1 (0.43, 1.95) | 10.6 (9.7, 11.7) |
|  | **18 hours** | 785 | 1.04 (0.93, 1.16) p = 0.472 | 1.31 (0.56, 2.44) | 10.2 (9.3, 11.3) |
|  | **24 hours** | 761 | 1.05 (0.95, 1.16) p = 0.31 | 1.58 (0.75, 2.94) | 10.1 (9.4, 11.2) |
|  | **30 hours** | 717 | 1.06 (0.97, 1.16) p = 0.18 | 1.73 (0.78, 3.46) | 9.7 (8.9, 10.8) |
|  | **36 hours** | 689 | 1.07 (0.99, 1.15) p = 0.082 | 2.11 (0.89, 3.69) | 9.9 (9.1, 11.2) |
|  | **42 hours** | 661 | 1.06 (0.99, 1.14) p = 0.087 | 2.29 (1, 4.06) | 10.15 (9.3, 11.4) |
|  | **48 hours** | 628 | 1.06 (1, 1.14) p = 0.057 | 2.57 (1.11, 4.5) | 9.8 (9.1, 10.8) |
