# Supplementary material for: Association of fluid balance with mortality in sepsis is modified by admission hemoglobin levels: A large database study
Source: PLoS One. 2021 Jun 14;16(6):e0252629. doi: 10.1371/journal.pone.0252629 (PMC8202933; doi:10.1371/journal.pone.0252629)
Supplement: S4 Table — Missing data of vital signs and laboratory tests were imputed with their median values. (DOCX) [file pone.0252629.s009.docx]

**S4 Table. Missing data values.** Missing data of vital signs and lab tests were imputed with their median values

|  | **Missing count** | **Missing proportion** |
| --- | --- | --- |
| **Lactate** | 1931 | 0.2375 |
| **Weight** | 721 | 0.0887 |
| **Respiratory rate** | 3 | 0.0004 |
| **Temperature** | 3 | 0.0004 |
| **Heart rate** | 1 | 0.0001 |
| **Minimum Glasgow Coma Scale score** | 1 | 0.0001 |
| **Creatinine** | 1 | 0.0001 |
| **White Blood Cell count** | 1 | 0.0001 |
