# Supplementary material for: Association of fluid balance with mortality in sepsis is modified by admission hemoglobin levels: A large database study
Source: PLoS One. 2021 Jun 14;16(6):e0252629. doi: 10.1371/journal.pone.0252629 (PMC8202933; doi:10.1371/journal.pone.0252629)
Supplement: S1 File — (DOCX) [file pone.0252629.s010.docx]

**S1 File. 28-day mortality at different hemoglobin percentage change and fluid balance change.**

**S1 Table 1. 28-day mortality at different hemoglobin percentage change and fluid balance change.**

| Patients with moderate anemia | | | | | |
| --- | --- | --- | --- | --- | --- |
|  | | | **Hemoglobin change** | | |
|  |  |  | **Decreasing** | **Stable** | **Increasing** |
|  |  |  | **Range** [-52.58%, -2.5%]  **Median [IQR]**  -9.38% [-14.29%, -5.81%] | **Range** [-2.5%, 2.5%]  **Median [IQR]**  0[0,0] | **Range** [2.5%, 91.98%]  **Median [IQR]**  10.84% [6%, 19.54%] |
| Fluid balance | **Group1** | **Range** [-4.3L, -0.46L]  **Median [IQR]** -1.29[-2.01, -0.85] | 11.69 | 11.30 | 18.24 |
|  | **Group2** | **Range** [-0.46L, 1.37L]  **Median [IQR]** 0.36[-0.06, 0.84] | 19.64 | 17.99 | 18.90 |
|  | **Group3** | **Range** [1.37L, 10.82L]  **Median [IQR]** 3.01[2.07, 4.49] | 29.25 | 26.44 | 20.95 |

** 28-day mortality at different fluid balance tertile and group of hemoglobin change in 24 hours for moderate anemia patients. Fluid balance and hemoglobin change% are stratified by the tertile of their values.*

**S1 Table 2. Results of Interaction analysis with differences analysed using Fisher’s Exact Test.**

| Patients with moderate anemia | | | | | |
| --- | --- | --- | --- | --- | --- |
|  | | | **Hemoglobin change** | | |
|  |  |  | **Decreasing** | **Stable** | **Increasing** |
|  |  |  | **Range** [-52.58%, -2.5%]  **Median [IQR]**  -9.38% [-14.29%, -5.81%] | **Range** [-2.5%, 2.5%]  **Median [IQR]**  0[0,0] | **Range** [2.5%, 91.98%]  **Median [IQR]**  10.84% [6%, 19.54%] |
| Fluid balance | **Group1** | **Range** [-4.3L, -0.46L]  **Median [IQR]** -1.29[-2.01, -0.85] | <0.001 | <0.001 | 0.009 |
|  | **Group2** | **Range** [-0.46L, 1.37L]  **Median [IQR]** 0.36[-0.06, 0.84] | 0.005 | <0.001 | 0.015 |
|  | **Group3** | **Range** [1.37L, 10.82L]  **Median [IQR]** 3.01[2.07, 4.49] | - | 0.471 | 0.034 |

** P value of Fisher’s Exact Test for moderate anemia patients at 24 hours.*
