# Supplementary material for: Association of fluid balance with mortality in sepsis is modified by admission hemoglobin levels: A large database study
Source: PLoS One. 2021 Jun 14;16(6):e0252629. doi: 10.1371/journal.pone.0252629 (PMC8202933; doi:10.1371/journal.pone.0252629)
Supplement: S2 File — (DOCX) [file pone.0252629.s011.docx]

**S2 File.** **Sensitivity analyses results after Propensity Score Matching.**

**Method description**

Propensity score matching was utilized to create a balanced cohort. We took fluid balance as a dichotomous(positive or negative) variable, and the probability of having a positive or negative fluid balance was estimated with baseline covariates. The nearest neighbor matching algorithm was adopted with a caliper size of 0.1. The package *matchit* in R was deployed for modeling.

After matching, the covariate balance was examined in the love plot. Finally, a total of 6,190 patients were included in further analysis.

**S2 Fig 1. Covariate balance measured by standardized mean difference**

**S2 Fig 1. Covariate balance measured by standardized mean difference.** The love plot for assessing the covariate balance before and after adjusting. Abbreviations: BP= Blood Pressure; ESKD: End Stage Kidney Disease; GCS = Glasgow Coma Scale; SOFA = Sequential Organ Failure Assessment; WBC = White Blood Cell

**S2 Fig 2. Visualization of regression results after propensity score matching**

1. **Moderate anemia patients b. Patients without moderate anemia**

**S2 Fig 2. Visualization of regression results after propensity score matching.** Logistic regression results for cohort after propensity score matching. The ORs represent the risk of 28-day mortality, with positive fluid balance at different observation windows after ICU admission. (a) and (b) show the regression results for patients with and without moderate anemia, respectively. Patients without moderate anemia had decreased risk of 28-day mortality with increasing fluid balance (OR 0.94, 95% CI 0.89 - 0.99 at 12 hours, p = 0.026). In moderate anemia patients, there was an increased risk of 28-day mortality (OR 1.08, 95% CI 1.02 - 1.14at 12 hours, p = 0.006) with increasing fluid balance. Abbreviations: FB = Fluid balance; ICU = Intensive Care Unit; OR = Odds Ratio

**S2 Table 1. Regression results after propensity score matching**

| Subgroup | Observation window | Patient Number | OR (95% CI) | Median fluid balance for Positive fluid balance, L (Median [IQR]) | Median hemoglobin for positive fluid balance, g/dL (Median [IQR]) |
| --- | --- | --- | --- | --- | --- |
| Moderate anemia patients | **6 hours** | 1869 | 1.04 (0.92, 1.18) p = 0.529 | 0.74 (0.31, 1.34) | 8.9 (8, 9.7) |
|  | **12 hours** | 1867 | 1.03 (0.95, 1.12) p = 0.46 | 0.96 (0.42, 1.97) | 8.8 (8, 9.5) |
|  | **18 hours** | 1860 | 1.06 (0.99, 1.13) p = 0.076 | 1.28 (0.56, 2.63) | 9 (8.2, 9.7) |
|  | **24 hours** | 1789 | 1.08 (1.02, 1.14) p = 0.006 | 1.59 (0.72, 3.03) | 8.8 (8.3, 9.5) |
| Patients without moderate anemia | **6 hours** | 4321 | 0.92 (0.84, 1) p = 0.053 | 0.72 (0.34, 1.33) | 10.9 (9.8, 12.2) |
|  | **12 hours** | 4318 | 0.94 (0.89, 0.99) p = 0.026 | 1.1 (0.46, 2.08) | 10.9 (9.97, 12.1) |
|  | **18 hours** | 4305 | 0.97 (0.93, 1.02) p = 0.214 | 1.35 (0.59, 2.71) | 10.7 (9.8, 11.9) |
|  | **24 hours** | 4201 | 1 (0.96, 1.03) p = 0.86 | 1.62 (0.69, 3.13) | 10.6 (9.6, 11.7) |

**S2 Table 2. Regression results of subgroups after propensity score matching**

| Subgroup | Observation window | Patient Number | OR (95% CI) | Median fluid balance for Positive fluid balance, L (Median [IQR]) | Median hemoglobin for positive fluid balance, g/dL (Median [IQR]) |
| --- | --- | --- | --- | --- | --- |
| All patients | **6 hours** | 6190 | 0.95 (0.88, 1.02) p = 0.146 | 0.72 (0.33, 1.34) | 10.4 (9.1, 11.8) |
|  | **12 hours** | 6185 | 0.96 (0.92, 1.01) p = 0.106 | 1.04 (0.45, 2.07) | 10.4 (9.3, 11.7) |
|  | **18 hours** | 6165 | 1 (0.96, 1.03) p = 0.854 | 1.31 (0.58, 2.68) | 10.3 (9.3, 11.53) |
|  | **24 hours** | 5990 | 1.02 (0.99, 1.05) p = 0.227 | 1.61 (0.7, 3.11) | 10.2 (9.1, 11.3) |
| Congestive heart failure patients | **6 hours** | 1494 | 0.99 (0.86, 1.13) p = 0.856 | 0.68 (0.3, 1.27) | 10.3 (9, 11.5) |
|  | **12 hours** | 1494 | 0.99 (0.9, 1.08) p = 0.836 | 1.01 (0.47, 1.94) | 10.3 (9.2, 11.4) |
|  | **18 hours** | 1488 | 1.02 (0.95, 1.09) p = 0.551 | 1.28 (0.65, 2.59) | 10.25 (9.33, 11.5) |
|  | **24 hours** | 1444 | 1.04 (0.98, 1.1) p = 0.221 | 1.44 (0.72, 2.92) | 9.8 (9.1, 10.9) |
| Moderate anemia patients with Congestive heart failure | **6 hours** | 518 | 1.04 (0.82, 1.3) p = 0.757 | 0.67 (0.26, 1.15) | 8.8 (7.95, 9.7) |
|  | **12 hours** | 518 | 1.01 (0.86, 1.18) p = 0.917 | 0.91 (0.41, 1.73) | 8.8 (8.3, 9.4) |
|  | **18 hours** | 517 | 1.06 (0.93, 1.2) p = 0.356 | 1.14 (0.56, 2.33) | 9 (8.5, 9.7) |
|  | **24 hours** | 491 | 1.1 (0.99, 1.22) p = 0.07 | 1.27 (0.7, 2.83) | 9.1 (8.6, 9.62) |
| Without moderate anemia, with Congestive heart failure | **6 hours** | 976 | 0.98 (0.81, 1.17) p = 0.803 | 0.69 (0.34, 1.34) | 10.9 (10.1, 12) |
|  | **12 hours** | 976 | 0.98 (0.87, 1.1) p = 0.745 | 1.12 (0.54, 2) | 10.8 (9.9, 11.9) |
|  | **18 hours** | 971 | 1 (0.92, 1.09) p = 0.938 | 1.35 (0.66, 2.62) | 10.8 (9.9, 11.9) |
|  | **24 hours** | 953 | 1.01 (0.94, 1.08) p = 0.819 | 1.57 (0.73, 2.93) | 10.3 (9.3, 11.4) |
| All patients with mechanical ventilation | **6 hours** | 853 | 0.91 (0.77, 1.06) p = 0.247 | 0.84 (0.38, 1.68) | 10.8 (9.7, 12.1) |
|  | **12 hours** | 853 | 0.96 (0.86, 1.06) p = 0.422 | 1.23 (0.46, 2.45) | 10.3 (9.2, 11.9) |
|  | **18 hours** | 852 | 0.98 (0.9, 1.07) p = 0.687 | 1.62 (0.7, 3.19) | 10.35 (9.38, 11.9) |
|  | **24 hours** | 848 | 0.99 (0.93, 1.06) p = 0.858 | 1.96 (0.9, 3.53) | 10.6 (9.3, 11.95) |
| Moderate anemia patients with mechanical ventilation | **6 hours** | 239 | 0.77 (0.55, 1.04) p = 0.096 | 0.9 (0.41, 1.57) | 9.25 (8.6, 9.8) |
|  | **12 hours** | 239 | 0.88 (0.71, 1.08) p = 0.232 | 1.08 (0.42, 2.33) | 9.1 (8.33, 9.78) |
|  | **18 hours** | 238 | 0.92 (0.77, 1.08) p = 0.304 | 1.71 (0.81, 3.38) | 9.2 (8.5, 9.7) |
|  | **24 hours** | 236 | 0.95 (0.82, 1.09) p = 0.453 | 2.21 (0.97, 3.81) | 8.5 (8.15, 8.8) |
| Without moderate anemia, with mechanical ventilation | **6 hours** | 614 | 0.99 (0.81, 1.21) p = 0.95 | 0.81 (0.38, 1.78) | 11.5 (10.4, 12.6) |
|  | **12 hours** | 614 | 1.01 (0.88, 1.15) p = 0.901 | 1.29 (0.48, 2.54) | 10.9 (9.8, 12.2) |
|  | **18 hours** | 614 | 1.02 (0.92, 1.13) p = 0.652 | 1.6 (0.66, 3.17) | 11 (9.83, 12.1) |
|  | **24 hours** | 612 | 1.02 (0.94, 1.11) p = 0.644 | 1.94 (0.83, 3.43) | 11.1 (9.67, 12.12) |
| All patients without mechanical ventilation | **6 hours** | 5337 | 0.96 (0.88, 1.03) p = 0.259 | 0.71 (0.32, 1.3) | 10.3 (9, 11.7) |
|  | **12 hours** | 5332 | 0.96 (0.91, 1.01) p = 0.128 | 1.01 (0.44, 2) | 10.4 (9.3, 11.7) |
|  | **18 hours** | 5313 | 1 (0.96, 1.04) p = 0.901 | 1.27 (0.57, 2.59) | 10.3 (9.3, 11.5) |
|  | **24 hours** | 5142 | 1.02 (0.99, 1.06) p = 0.187 | 1.55 (0.66, 3.03) | 10.2 (9.1, 11.3) |
| Moderate anemia patients without mechanical ventilation | **6 hours** | 1630 | 1.13 (0.98, 1.29) p = 0.089 | 0.7 (0.3, 1.31) | 8.9 (8, 9.6) |
|  | **12 hours** | 1628 | 1.08 (0.98, 1.18) p = 0.12 | 0.94 (0.42, 1.89) | 8.7 (7.93, 9.4) |
|  | **18 hours** | 1622 | 1.1 (1.03, 1.19) p = 0.008 | 1.22 (0.53, 2.5) | 8.9 (8.2, 9.7) |
|  | **24 hours** | 1553 | 1.12 (1.05, 1.2) p < 0.001 | 1.52 (0.67, 2.9) | 8.9 (8.3, 9.6) |
| Without moderate anemia, without mechanical ventilation | **6 hours** | 3707 | 0.9 (0.81, 0.99) p = 0.032 | 0.71 (0.34, 1.3) | 10.8 (9.6, 12.2) |
|  | **12 hours** | 3704 | 0.92 (0.87, 0.98) p = 0.01 | 1.06 (0.46, 2.04) | 10.9 (10, 12.1) |
|  | **18 hours** | 3691 | 0.96 (0.91, 1.01) p = 0.094 | 1.3 (0.59, 2.61) | 10.65 (9.8, 11.8) |
|  | **24 hours** | 3589 | 0.99 (0.95, 1.03) p = 0.608 | 1.57 (0.66, 3.09) | 10.5 (9.6, 11.7) |
| All patients with chronic kidney disease | **6 hours** | 970 | 0.83 (0.64, 1.05) p = 0.126 | 0.62 (0.28, 1.08) | 9.85 (8.7, 11) |
|  | **12 hours** | 968 | 0.95 (0.82, 1.1) p = 0.526 | 0.92 (0.4, 1.76) | 9.8 (8.9, 11.1) |
|  | **18 hours** | 962 | 1.01 (0.9, 1.13) p = 0.802 | 1.13 (0.47, 2.3) | 10 (9.03, 11.07) |
|  | **24 hours** | 925 | 1.05 (0.96, 1.15) p = 0.308 | 1.32 (0.57, 2.77) | 9.8 (9.2, 10.75) |
| Moderate anemia patients with chronic kidney disease | **6 hours** | 384 | 0.78 (0.49, 1.18) p = 0.256 | 0.56 (0.26, 0.97) | 8.75 (8, 9.43) |
|  | **12 hours** | 383 | 0.96 (0.75, 1.21) p = 0.754 | 0.83 (0.38, 1.69) | 8.7 (8.12, 9.2) |
|  | **18 hours** | 380 | 1.04 (0.86, 1.25) p = 0.679 | 1.06 (0.44, 2.06) | 8.65 (7.95, 9.85) |
|  | **24 hours** | 361 | 1.08 (0.92, 1.25) p = 0.355 | 1.14 (0.53, 2.66) | 9.1 (8.65, 9.67) |
| Without moderate anemia, with chronic kidney disease | **6 hours** | 586 | 0.87 (0.63, 1.16) p = 0.358 | 0.65 (0.3, 1.19) | 10.5 (9.33, 11.6) |
|  | **12 hours** | 585 | 0.96 (0.78, 1.15) p = 0.644 | 0.95 (0.42, 1.8) | 10.8 (9.7, 11.75) |
|  | **18 hours** | 582 | 1 (0.86, 1.15) p = 0.985 | 1.24 (0.5, 2.37) | 10.3 (9.67, 11.3) |
|  | **24 hours** | 564 | 1.03 (0.91, 1.16) p = 0.6 | 1.46 (0.64, 2.9) | 10.15 (9.4, 11.2) |
