# Supplementary material for: Association of fluid balance with mortality in sepsis is modified by admission hemoglobin levels: A large database study
Source: PLoS One. 2021 Jun 14;16(6):e0252629. doi: 10.1371/journal.pone.0252629 (PMC8202933; doi:10.1371/journal.pone.0252629)
Supplement: S3 File — (DOCX) [file pone.0252629.s012.docx]

**S3 File. Sensitivity analyses for different definitions of moderate anemia.**

**S3 Fig 1. Visualization of sensitivity analyses results for moderate anemia defined as Hb 7-9g/dL**

1. **Moderate anemia patients b. Patients without moderate anemia**

**S3 Fig 1. Visualization of sensitivity analyses results for moderate anemia defined as Hb 7-9g/dL.** Logistic regression results for sepsis patients, where moderate anemia was defined as admission Hb 7-9 g/dL. The ORs represent the risk of 28-day mortality at different observation windows after ICU admission. (a) and (b) show the regression results with and without moderate anemia, respectively. Moderate anemia patients had no significant difference in 28-day mortality (OR 1.06, 95% CI 0.99 – 1.13, p=0.07 at 24 hours) with increasing fluid balance. Conversely, patients without moderate anemia had decreased risk of 28-day mortality (OR 0.9, 95% CI 0.85 – 0.96, p = 0.01 at 6 hours; OR 0.95, 95% CI 0.91 – 0.99, p = 0.008 at 12 hours) with increasing fluid balance. Abbreviations: FB = Fluid balance; ICU = Intensive Care Unit; OR = Odds Ratio

**S3 Table 1. Sensitivity analyses results for moderate anemia defined as Hb 7-9g/dL**

| Subgroup | Observation window | Patient Number | OR (95% CI) | Median fluid balance, L (Median [IQR]) | Median hemoglobin, g/dL (Median [IQR]) |
| --- | --- | --- | --- | --- | --- |
| All patients | **6 hours** | 8132 | 0.93 (0.87, 0.98) p = 0.008 | 0.79 (0.36, 1.49) | 10.3 (8.9, 11.7) |
|  | **12 hours** | 8126 | 0.96 (0.92, 0.99) p = 0.022 | 1.18 (0.5, 2.33) | 10.3 (9.2, 11.6) |
|  | **18 hours** | 8103 | 0.99 (0.96, 1.02) p = 0.502 | 1.5 (0.67, 3.01) | 10.2 (9.2, 11.5) |
|  | **24 hours** | 7883 | 1.01 (0.98, 1.03) p = 0.586 | 1.82 (0.8, 3.47) | 10.2 (9.1, 11.4) |
| Moderate anemia patients | **6 hours** | 1207 | 1.05 (0.92, 1.2) p = 0.475 | 0.85 (0.37, 1.53) | 8.4 (7.6, 9) |
|  | **12 hours** | 1207 | 1.03 (0.94, 1.13) p = 0.512 | 1.25 (0.52, 2.38) | 8.2 (7.6, 8.9) |
|  | **18 hours** | 1204 | 1.05 (0.97, 1.13) p = 0.195 | 1.5 (0.71, 3.02) | 8.3 (7.5, 9.12) |
|  | **24 hours** | 1169 | 1.06 (0.99, 1.13) p = 0.07 | 1.76 (0.78, 3.45) | 8.2 (7.6, 8.88) |
| Patients without moderate anemia | **6 hours** | 6925 | 0.9 (0.85, 0.96) p = 0.001 | 0.78 (0.36, 1.47) | 10.6 (9.3, 11.9) |
|  | **12 hours** | 6919 | 0.95 (0.91, 0.99) p = 0.008 | 1.17 (0.5, 2.32) | 10.6 (9.5, 11.8) |
|  | **18 hours** | 6899 | 0.98 (0.95, 1.01) p = 0.238 | 1.5 (0.67, 3) | 10.3 (9.4, 11.6) |
|  | **24 hours** | 6714 | 1 (0.97, 1.03) p = 0.917 | 1.83 (0.8, 3.48) | 10.3 (9.3, 11.5) |

**S3 Fig 2. Visualization of sensitivity analyses results for moderate anemia defined as Hb 7-8g/dL**

1. **Moderate anemia patients b. Patients without moderate anemia**

**S3 Fig 2. Visualization of sensitivity analyses results for moderate anemia defined as Hb 7-8g/dL.** Logistic regression results for sepsis patients, where moderate anemia was defined as admission Hb 7-8g/dL. The ORs represent the risk of 28-day mortality at different observation windows after ICU admission. (a) and (b) show the regression results with and without moderate anemia, respectively. Moderate anemia patients had no significant difference in mortality (OR 1.07, 95% CI 0.96 – 1.2, p = 0.211 at 24 hours) with increasing fluid balance. In contrast, patients without moderate anemia had decreased risk of 28-day mortality (OR 0.92, 95% CI 0.87 – 0.98, p = 0.006 at 6 hours; OR 0.95, 95% CI 0.92 – 0.99, p = 0.011 at 12 hours) with increasing fluid balance. Abbreviations: FB = Fluid balance; ICU = Intensive Care Unit; OR = Odds Ratio

**S3 Table 2. Sensitivity analyses results for moderate anemia defined as Hb 7-8g/dL**

| Subgroup | Observation window | Patient Number | OR (95% CI) | Median fluid balance, L (Median [IQR]) | Median hemoglobin, g/dL (Median [IQR]) |
| --- | --- | --- | --- | --- | --- |
| All patients | **6 hours** | 8132 | 0.93 (0.87, 0.98) p = 0.008 | 0.79 (0.36, 1.49) | 10.3 (8.9, 11.7) |
|  | **12 hours** | 8126 | 0.96 (0.92, 0.99) p = 0.022 | 1.18 (0.5, 2.33) | 10.3 (9.2, 11.6) |
|  | **18 hours** | 8103 | 0.99 (0.96, 1.02) p = 0.502 | 1.5 (0.67, 3.01) | 10.2 (9.2, 11.5) |
|  | **24 hours** | 7883 | 1.01 (0.98, 1.03) p = 0.586 | 1.82 (0.8, 3.47) | 10.2 (9.1, 11.4) |
| Moderate anemia patients | **6 hours** | 428 | 1.01 (0.81, 1.26) p = 0.911 | 0.9 (0.41, 1.53) | 7.7 (7.3, 8.7) |
|  | **12 hours** | 428 | 1.04 (0.89, 1.2) p = 0.622 | 1.31 (0.5, 2.46) | 7.8 (7.3, 8.6) |
|  | **18 hours** | 427 | 1.06 (0.93, 1.2) p = 0.364 | 1.61 (0.66, 2.94) | 7.8 (7.07, 9.03) |
|  | **24 hours** | 409 | 1.07 (0.96, 1.2) p = 0.211 | 1.77 (0.85, 3.41) | 8 (7.65, 8.85) |
| Patients without moderate anemia | **6 hours** | 7704 | 0.92 (0.87, 0.98) p = 0.006 | 0.78 (0.36, 1.49) | 10.4 (9.1, 11.8) |
|  | **12 hours** | 7698 | 0.95 (0.92, 0.99) p = 0.011 | 1.17 (0.5, 2.32) | 10.4 (9.3, 11.7) |
|  | **18 hours** | 7676 | 0.98 (0.96, 1.02) p = 0.328 | 1.5 (0.67, 3.01) | 10.3 (9.2, 11.5) |
|  | **24 hours** | 7474 | 1 (0.98, 1.03) p = 0.864 | 1.82 (0.8, 3.48) | 10.2 (9.1, 11.4) |
