# Supplementary material for: Association of fluid balance with mortality in sepsis is modified by admission hemoglobin levels: A large database study
Source: PLoS One. 2021 Jun 14;16(6):e0252629. doi: 10.1371/journal.pone.0252629 (PMC8202933; doi:10.1371/journal.pone.0252629)
Supplement: S4 File — (DOCX) [file pone.0252629.s013.docx]

**S4 File. Description of fluid balance**

**S4 Fig. Distribution of fluid balance at 24 hours after ICU admission**

**S10 Fig. Distribution of fluid balance at 24 hours after ICU admission.** Histogram for 24-hour fluid balance. X-axis represents the fluid balance value(L), y-axis represents the number of patients.

**S4 Table. Description of positive and negative fluid balance at 24 hours after ICU admission**

|  | Range | Median [IQR] |
| --- | --- | --- |
| Negative fluid balance | [-4.41, 0] | -1.14[-1.91, -0.57] |
| Positive fluid balance | [0.001, 10.83] | 1.79[0.78, 3.43] |
