# Supplementary material for: Association of fluid balance with mortality in sepsis is modified by admission hemoglobin levels: A large database study
Source: PLoS One. 2021 Jun 14;16(6):e0252629. doi: 10.1371/journal.pone.0252629 (PMC8202933; doi:10.1371/journal.pone.0252629)
Supplement: Attachment — Submitted filename: Response to Reviewers.docx [file pone.0252629.s014.docx]

May 14, 2021

Dear Reviewers,

Response to Reviewers: Association of fluid balance with mortality in sepsis is modified by admission hemoglobin levels: a large database study

We sincerely thank you for your expert review of our manuscript. Based on your review, we have done an extensive re-analysis by changing the variable of fluid balance from a dichotomous variable to continuous variable. Additionally, we have adjusted our inclusion and exclusion criteria to avoid extreme outliers and thus improve accuracy of our results. After re-analysis, the results are in keeping with the trend of results of our original analysis. Our conclusions as well as clinical implications of our research remain consistent with our original manuscript.

Do find our responses to your comments and concerns below. To allow a cohesive response, we have grouped some of the related comments together.

**Reviewer #1**

**Major Concerns**

***1. Hemoglobin concentrations are referred to as "admission values", but time points for blood sampling are not specified. Page 11, lines 197-198 exclusion criteria: "174 without hemoglobin measurements in the first 3 days", does that mean that admission hemoglobin value is analyzed sometime during the first 72 h? Please, clarify as it seems important in order to be able to interpret results. Add to/modify accordingly in the Methods, (Results), Discussion.***

Response

**Regarding specification of time points for blood sampling and whether admission hemoglobin value is analyzed some time during the first 72 hours:** We have examined the timing of admission hemoglobin measurements as suggested. As shown in the below figure (***S1 Fig***), most patients have their first hemoglobin measured at admission: 7,577(93.2%) patients had their first hemoglobin measured within 6 hours after ICU admission. 7,946(97.7%) patients had their first hemoglobin measured within 12 hours. We give a detailed description of hemoglobin in ***S1 Fig***, including the distribution of admission hemoglobin (***S1 Fig a***), the timing of admission hemoglobin (***S1 Fig b***), distribution of times at which hemoglobin was measured (***S1 Fig c***), and also the distribution of hemoglobin percentage change (***S1 Fig d***).

**Add to/modify accordingly in the Methods, (Results), Discussion:** The relevant descriptions have also been included in the relevant (Lines 323-325) sections of our manuscript.

**S1 Fig. Distribution of hemoglobin measurements**

**S1 Fig. Distribution of hemoglobin measurements.** (a) Distribution of Hb measurements. (b) Time of Hb measurement after ICU admission (Hours). (c) Number of Hb measurements per patient in the first 24 hours after ICU admission. (d) Distribution of Hb change (%) in the first 24 hours after ICU admission. Hb change (%) was calculated as (*admission Hb- last Hb in 24 hours)/admission Hb*, where Hb measures after blood transfusion were not taken into consideration. Abbrieviations: Hb = hemoglobin.

***2. p 16, line 261. The choice to present data in tertiles could be discussed and explained. The spread within tertiles is wide.***

***3. p 8, line 140 - 141 "Total fluid balance was winsorized at the 0.5 and 99.5 percentiles to limit the effect of extreme values", Please, give rational for why are data winsorized?***

***4i. Figure 3a. tertile 1 has a negative fluid balance with the range -31,25 - 0,61. The authors might wish to discuss and explain how such an extreme negative fluid balance is possible in early sepsis and how these data should be interpreted.***

***5i. The spread/variance of fluid balance over the first 24 hours as depicted in Figure 3a seems extreme. How do the investigators explain negative fluid balance of 32 L?***

Response

**Questions #2 #3 #4:** We thoroughly examined the fluid balance records for our cohort and found that the wide range of negative fluid balance was caused by erroneous genitourinary fluid records (some input urinary bladder irrigation records were recorded as output, causing extreme negative fluid balance volume). To avoid the error records (extreme values), we removed these records and also amended the threshold for extracting fluid records**.** For the same reason, we amended the winsorization window to 1 and 99 percentiles **(**0.5 and 99.5 percentiles in previous analyses**)** to further reduce the effect of possibly spurious outliers**.** Thereafter, the spread of fluid balance retracted was found to be in a clinically feasible range ([-4.41L, 10.83L] for 24 hours fluid balance). The detailed description of fluid balance distribution is added to the manuscript and supplementary material (***Fig S10a and Table S10b***).

**S10a. Distribution of fluid balance at 24 hours after ICU admission**

**S10a.** Histogram for 24-hour fluid balance. X-axis represents the fluid balance value(L), y-axis represents the number of patients.

**S10b. Description of positive and negative fluid balance at 24 hours after ICU admission**

|  | Range | Median [IQR] |
| --- | --- | --- |
| Negative fluid balance | [-4.41, 0] | -1.14[-1.91, -0.57] |
| Positive fluid balance | [0.001, 10.83] | 1.79[0.78, 3.43] |

**Regarding the choice to present fluid balance data in tertiles:** Fluid balance data were presented in tertiles in order to allow for a balanced group size for each subgroup. When attempting to analyze fluid balance in quartiles, we found that the number of patients in each subgroup was not balanced (1 group had less than 100 patients). Stratifying fluid balance by tertile would allow for more balanced cohort sizes and thus more reliable results.

***4ii. In fig 3b range of hemoglobin change in the third tertile (with increasing hemoglobin) is 5 - 285,7 %. This does seem peculiar as the group was defined as non-anemic. Are data correctly reported?***

Response

Thanks for pointing out the unusually wide range of hemoglobin changes in our original manuscript. The wide range was caused by outlier values. We have newly set an upbound threshold of 21 g/dL to avoid these extreme values. In our revised results, overall distribution of hemoglobin change% is [-52.58%, 91.89%] in tertile 1 and [2.5%, 91.89%] in tertile 3(***Shown in Fig 3***)***.*** (Distribution figure is shown in response to question #1 – ***S1 Fig d***)

**Fig 3. 3D-bar plots showing 28-day mortality for different fluid balance and hemoglobin in patients with moderate anemia**

**Fig 3.** 3D-bar plots showing 28-day mortality for different fluid balance and hemoglobin change% in patients with moderate anemia. X-axis represents hemoglobin change, y-axis represents fluid balance and z-axis represents mortality at 28 days. For each graph, the pillar in dark red (dotted) represents the highest mortality. The grey pillars represent interactions with lower mortality (p<0.05). Pink pillar (striped) represents interactions in which no statistically significant difference from the highest mortality interaction was found. Mortality risk was highest when haemoglobin decreased in patients with moderate anemia who had the most positive fluid balance. Hemoglobin change% was calculated as 100 * (last hemoglobin record - first hemoglobin record) / first hemoglobin record. Fluid balance is stratified by the tertile of its value. Hemoglobin change was stratified to three groups: decreasing (< -2.5%), stable (±2.5%) and increasing (>2.5%). Abbreviations: FB = Fluid balance; Hb = hemoglobin;

***5ii. p9, line 157-159: "Total fluid balance during ICU stay was summarized as a continuous variable in baseline statistics and treated as a dichotomous variable (positive fluid balance versus negative fluid balance) in further modelling." Please, give rational for dichotomizing the data.***

Response

In the previous analyses, we summarized fluid balance as a continuous variable in ***Table 1*** to describe its distribution. We had taken fluid balance as a dichotomous variable in our multivariable logistic regression (positive or negative) analyses.

Our revised analysis has been inspired by your question. We have now reperformed all regression analyses with fluid balance as a continuous variable in order to look at the effect of fluid balance volume.

The revised results are consistent with our prior findings. Additionally, the interpretation of these revised analyses will be more applicable to clinical practice. The main results are:

For moderate anemia patients, increasing fluid balance was associated with increased risk of 28-day mortality at 24 hours after ICU admission (OR 1.05, 95% CI 1.01 - 1.1, p = 0.022) with median fluid balance of 1.78 L (IQR 0.78L - 3.41L). ). Median hemoglobin level overall showed a slight decrease from 8.8g/dL (IQR 7.9g/dL - 9.6g/dL) at 6 hours after ICU admission, to 8.7g/dL at 24 hours after ICU admission (median IQR 8.1g/dL - 9.4g/dL). For patients without moderate anemia, increasing fluid balance was associated with decreased risk of 28-day mortality at 6 hours (OR 0.91, 95% CI 0.84 - 0.97, p=0.005) and 12 hours (OR 0.94, 95% CI 0.9 - 0.98, p=0.007) after ICU admission (Fig 2b, Table 2). Median fluid balance was 0.78L (IQR 0.38L - 1.49L) at 6 hours and 1.19L (IQR 0.5L - 2.33L) at 12 hours after ICU admission. Median hemoglobin level showed a decrease from 10.9g/dL (IQR 9.7g/dL – 12.2g/dL) at 6 hours after ICU admission, to 10.6g/dL (IQR 9.6g/dL – 11.8g/dL) at 24 hours after ICU admission (***Fig 2 and Table 2***).

**Fig 2. Risk of 28-day mortality for sepsis patients at different observation windows after ICU admission**

**Fig 2.** Risk of 28-day mortality for sepsis patients at different observation windows after ICU admission. (a) and (b) are for patients with and without moderate anemia respectively. Odds Ratio of each observation window represents the adjusted Odds Ratio of 28-day mortality derived from multivariable logistic regression. Abbreviations: FB = fluid balance; ICU = Intensive Care Unit; OR = Odds Ratio

**Table 2 Results of 28-day mortality for sepsis patients at different observation windows after ICU admission**

| Subgroup | Observation window | Patient Number | OR (95% CI) | Median fluid balance, L (Median [IQR]) | Median hemoglobin, g/dL (Median [IQR]) |
| --- | --- | --- | --- | --- | --- |
| Moderate anemia patients | 6 hours | 2469 | 0.98 (0.88, 1.08) p = 0.624 | 0.8 (0.35, 1.49) | 8.8 (7.9, 9.6) |
|  | 12 hours | 2466 | 1 (0.94, 1.07) p = 0.97 | 1.15 (0.5, 2.33) | 8.7 (8, 9.5) |
|  | 18 hours | 2459 | 1.04 (0.98, 1.09) p = 0.193 | 1.46 (0.66, 2.94) | 8.9 (8, 9.7) |
|  | 24 hours | 2375 | 1.05 (1.01, 1.1) p = 0.022 | 1.78 (0.78, 3.41) | 8.7 (8.1, 9.4) |
| Patients without moderate anemia | 6 hours | 5663 | 0.91 (0.84, 0.97) p = 0.005 | 0.78 (0.38, 1.49) | 10.9 (9.7, 12.2) |
|  | 12 hours | 5660 | 0.94 (0.9, 0.98) p = 0.007 | 1.19 (0.5, 2.33) | 10.9 (9.9, 12) |
|  | 18 hours | 5644 | 0.97 (0.94, 1.01) p = 0.108 | 1.53 (0.68, 3.03) | 10.6 (9.7, 11.8) |
|  | 24 hours | 5508 | 0.99 (0.96, 1.02) p = 0.405 | 1.84 (0.8, 3.52) | 10.6 (9.6, 11.8) |

The results of subgroups and sensitivity analyses are consistent with the prior findings and also support our previous conclusions. All related results are updated in the manuscript and additional files. We have summarized the results of our subgroup and sensitivity analyses in a newly added ***Table 3***.

**Table 3 Summary of findings in subgroup analyses and sensitivity analyses**

|  | Findings | Related file |
| --- | --- | --- |
| Subgroup analysis | - For patients with congestive heart failure, the results were in line with the main analyses for moderate anemia patients. In contrast, for patients without moderate anemia, there was no significant difference in risk of 28-day mortality. - For patients with mechanical ventilation on ICU admission, no statistically significant difference in risk of 28-day mortality was found for patients with or without moderate anemia. - For patients without mechanical ventilation on ICU admission, the results were in line with the main analyses for patients with and without moderate anemia. - For patients with chronic kidney disease, no statistically significant difference in risk of 28-day mortality was found for patients with or without moderate anemia. | **S3 Fig and Table** |
| Sensitivity analyses for patients without blood transfusion | In line with results of main analyses, magnitude of risk of 28-day mortality was higher in patients with moderate anemia compared to patients without moderate anemia. | **S4 Fig and Table** |
| Sensitivity analyses for extending observation window to 48 hours | Results were consistent with main analyses | **S5 Fig and Table** |
| Sensitivity analyses for imputation method | Results were consistent with main analyses | **S7 Fig** |
| Sensitivity analyses for propensity score matching | Results were consistent with main analyses | **S8 Fig and Table** |
| Sensitivity analyses for modified definitions of moderate anemia | In line with results of main analyses, with moderate anemia defined as both 7-8g/dL and 7-9g/dL, no significant difference in risk of 28-day mortality was found in moderate anemia patients. Moreover, decreased risk of 28-day mortality was found in patients without moderate anemia. | **S9 Fig and Table** |

***6. The manuscript might benefit from general revision of the language.***

Response

We have reviewed the language of our manuscript and made amendments where required.

**Minor concerns:**
***1. p 6, line 76 "It is associated with high mortality and morbidity in survivors" please reformulate***

Response

We have reviewed the above line and reformulated it as:

Sepsis involves a dysregulated inflammatory response to infection that leads to organ dysfunction, which is associated with high mortality and morbidity (1, 2).

***2. p 6, line 82, please comment further on the two references mentioned.***

Response

We have reviewed the above and made amendments in the manuscript as:

Two prior randomized trials - the FEAST trial in children (1) and the trial by Andrews *et al* in adults (2) demonstrated *increased* mortality with an early fluid resuscitation strategy. Both the FEAST and Andrews trials were conducted in patients who had moderate anemia (mean hemoglobin 7.1 g/dL in FEAST and mean hemoglobin 7.8 g/dL in the Andrews trial). Nonetheless, the results from both trials cannot be directly applied to ICU patients with lesser degrees of anemia. A subsequent analysis of the FEAST trial could not demonstrate an effect modification by anemia, likely due to the already low hemoglobin levels, showing uniformly deleterious effects of fluid balance across hemoglobin levels (3). Unlike the FEAST trial analysis, our large database has allowed us to investigate the effect of fluid balance both in patients with and without moderate anemia.

***3. p 6, line 94 "duration of ventilation, length of stay" please specify***

Response

We have reviewed the above line and made amendments in the manuscript as such:

A retrospective cohort study of the ARISE trial (4) found each litre of intravenous fluid administration was significantly but weakly associated with a reduction in haemoglobin concentration by 1-1.3g/dL at 24 hours and 72 hours of admission. Additionally, for each 1g/dL decrease in haemoglobin concentration during 72 hours of admission, duration of invasive ventilation, ICU stay and hospital length of stay were longer.

***4. p 7, line 110-111 "have poorer outcomes in the first 24 hours", please correct***

Response

We have reviewed the above line and made amendments in the manuscript as such:

We hypothesized that with increasing fluid balance in the first 24 hours of ICU admission, those with moderate anemia (hemoglobin 7-10 g/dL) would have higher risk of 28-day mortality compared to those without moderate anemia (hemoglobin > 10g/dL)

**Reviewer #2**

***1. We don't know in what clinical situation the patients were in when the “admission Hb” was taken. Had the patients already received i.v. fluid a bolus 30 mL/kg? Had they low MAP, which causes hemodilution by capillary refill? Had they waited a long time before entering the ICU? The analysis gets more complicated as Hb varies greatly among populations, and even within populations.***

Response

Most patients have their admission hemoglobin taken on ICU admission (7,577(93.2%) patients had their first Hb measured within 6 hours after ICU admission). To assess patients’ conditions at ICU admission, we summarized the baseline characteristics in ***Table 1***.

As shown in ***Table 1***, the Mean Arterial Pressure of both groups was not low on admission to the ICU (76mmHg [IQR 66mmHg - 88mmHg] for moderate anemia patients, 81mmHg [IQR 70mmHg - 94mmHg] for patients without moderate anemia).

We do acknowledge the limitation that our data does not include information on the waiting time before entering the ICU. It also lacks the information on fluid boluses received before ICU admission. Instead, our study focuses on changes in hemoglobin with respect to hemoglobin on admission to ICU.

Indeed, hemoglobin varies greatly within and between populations, however hemoglobin heterogeneity is not a major focus of this study. The study aims to investigate the effect of fluid balance on mortality in sepsis patients with respect to their hemoglobin level on admission to ICU, and we hypothesized that the effect would differ according to hemoglobin levels on admission to ICU. To narrow down, we defined a moderate anemia group at Hb 7-10 g/dL and a non- moderate anemia group at >10 g/dL, and the effect of increasing fluid balance was assessed for patients with and without moderate anemia respectively. The results showed that there was a decreased risk of 28-day mortality only for patients without moderate anemia, while for moderate anemia patients, increasing fluid balance at 24 hours was associated with significantly increased risk of 28-day mortality.

Inspired by your concern regarding hemoglobin heterogeneity within populations, we have performed further sensitivity analyses with modified definitions of moderate anemia (with moderate anemia defined as 7-8 g/dL and 7-9 g/dL). These findings are consistent with results of our main analysis – patients without moderate anemia had decreased risk of 28-day mortality with increasing fluid balance whereas patients with moderate anemia had no significant difference in risk of 28-day mortality with increasing fluid balance.

***2. If the authors can clearly point out the clinical implication of their results I am willing to change my judgment. Could it be that infusing much fluid on the first day of care is beneficial is Hb is high?***

Response

We aim to highlight that current guidelines for sepsis patients, which recommend large fluid boluses regardless of hemoglobin levels, may need to be relooked at. Admission hemoglobin levels should be an important consideration for future prospective trials of fluid therapy in patients with sepsis. We hypothesized that patients who have moderate anemia on ICU admission may have poorer outcomes with increasing fluid balance.

Indeed, our results have shown that an increasing fluid balance in patients without moderate anemia may be beneficial (OR 0.91, 95% CI 0.84 - 0.97, p=0.005 at 6 hours after ICU admission; OR 0.94, 95% CI 0.9 - 0.98, p=0.007 at 12 hours after ICU admission) (Shown in ***Fig 2 and Table 2***) – this is in line with current guideline recommendations. On the other hand, our results show an association of increasing fluid balance with an increased risk of 28-day mortality for patients with moderate anemia (OR 1.05, 95% CI 1.01 - 1.1, p = 0.022 at 24 hours after ICU admission). This elucidates a potential danger of fluid resuscitation in these patients with moderate anemia in particular.

**Fig 2. Risk of 28-day mortality for sepsis patients at different observation windows after ICU admission**

**Fig 2.** Risk of 28-day mortality for sepsis patients at different observation windows after ICU admission. (a) and (b) are for patients with and without moderate anemia respectively. Odds Ratio of each observation window represents the adjusted Odds Ratio of 28-day mortality derived from multivariable logistic regression. Abbreviations: FB = fluid balance; ICU = Intensive Care Unit; OR = Odds Ratio

**Table 2 Results of 28-day mortality for sepsis patients at different observation windows after ICU admission**

| Subgroup | Observation window | Patient Number | OR (95% CI) | Median fluid balance, L (Median [IQR]) | Median hemoglobin, g/dL (Median [IQR]) |
| --- | --- | --- | --- | --- | --- |
| Moderate anemia patients | 6 hours | 2469 | 0.98 (0.88, 1.08) p = 0.624 | 0.8 (0.35, 1.49) | 8.8 (7.9, 9.6) |
|  | 12 hours | 2466 | 1 (0.94, 1.07) p = 0.97 | 1.15 (0.5, 2.33) | 8.7 (8, 9.5) |
|  | 18 hours | 2459 | 1.04 (0.98, 1.09) p = 0.193 | 1.46 (0.66, 2.94) | 8.9 (8, 9.7) |
|  | 24 hours | 2375 | 1.05 (1.01, 1.1) p = 0.022 | 1.78 (0.78, 3.41) | 8.7 (8.1, 9.4) |
| Patients without moderate anemia | 6 hours | 5663 | 0.91 (0.84, 0.97) p = 0.005 | 0.78 (0.38, 1.49) | 10.9 (9.7, 12.2) |
|  | 12 hours | 5660 | 0.94 (0.9, 0.98) p = 0.007 | 1.19 (0.5, 2.33) | 10.9 (9.9, 12) |
|  | 18 hours | 5644 | 0.97 (0.94, 1.01) p = 0.108 | 1.53 (0.68, 3.03) | 10.6 (9.7, 11.8) |
|  | 24 hours | 5508 | 0.99 (0.96, 1.02) p = 0.405 | 1.84 (0.8, 3.52) | 10.6 (9.6, 11.8) |

**Reviewer #3**

***1i.The article lacks certain novelty, and the results need to be revised deeply; firstly, the amount of fluid balance is not clear in the definition of variables. The more positive fluid balance doses have a greater impact on the prognosis of patients. I will suggest that the author can make a stratified analysis between different fluid balance and different hemoglobin levels to clarify the influence of hemoglobin levels on the prognosis of ICU patients during fluid therapy.***

***2. The definition of moderate anemia with hemoglobin 7-10g/dl is not properly, for hemoglobin lower than 8g/dl may influence patients’ outcome. The author needs to stratify different levels of hemoglobin, not just roughly with 7-10g/dl.***

Response

**The amount of fluid balance is not clear in the definition of variables:**

With regard to the amount of fluid balance, we have newly included a more detailed description in ***Table 1*** and ***Fig S10a*** and ***Table S10b***. The median total fluid balance in the first 24 hours of ICU stay is 0.36L[IQR -0.84L - 2.07L] for moderate anemia patients, and 0.18L[IQR -1.10L - 1.94L] for patients without moderate anemia.

**S10a. Distribution of fluid balance at 24 hours after ICU admission**

**S10a.** Histogram for 24-hour fluid balance. X-axis represents the fluid balance value(L), y-axis represents the number of patients.

**S10b. Description of positive and negative fluid balance at 24 hours after ICU admission**

|  | Range | Median [IQR] |
| --- | --- | --- |
| Negative fluid balance | [-4.41, 0] | -1.14[-1.91, -0.57] |
| Positive fluid balance | [0.001, 10.83] | 1.79[0.78, 3.43] |

**Regarding the definition of moderate anemia with hemoglobin 7-10d/dL:** The reason for definition of moderate anemia as 7-10g/dL in our main analysis is that the 10g/dL cut-off would be in-line with the threshold for blood transfusion for early goal-directed therapy in sepsis (5, 6). We have done further analyses on different definitions of moderate anemia as seen below.

**The author can make a stratified analysis between different fluid balance and different hemoglobin levels to clarify the influence of hemoglobin levels on the prognosis of ICU patients during fluid therapy. The author needs to stratify different levels of hemoglobin, not just roughly with 7-10g/dL:** We have taken your suggestion and that of the other reviewers into consideration and have changed the fluid balance variable from a dichotomous variable to a continuous variable in our regression models. With this re-analysis, results are consistent with our prior findings. We believe that this will be more applicable to clinical practice. These results are seen below in ***Fig 2 and Table 2***.

**Fig 2. Risk of 28-day mortality for sepsis patients at different observation windows after ICU admission**

**Fig 2.** Risk of 28-day mortality for sepsis patients at different observation windows after ICU admission. (a) and (b) are for patients with and without moderate anemia respectively. Odds Ratio of each observation window represents the adjusted Odds Ratio of 28-day mortality derived from multivariable logistic regression. Abbreviations: FB = fluid balance; ICU = Intensive Care Unit; OR = Odds Ratio

**Table 2 Results of 28-day mortality for sepsis patients at different observation windows after ICU admission**

| Subgroup | Observation window | Patient Number | OR (95% CI) | Median fluid balance, L (Median [IQR]) | Median hemoglobin, g/dL (Median [IQR]) |
| --- | --- | --- | --- | --- | --- |
| Moderate anemia patients | 6 hours | 2469 | 0.98 (0.88, 1.08) p = 0.624 | 0.8 (0.35, 1.49) | 8.8 (7.9, 9.6) |
|  | 12 hours | 2466 | 1 (0.94, 1.07) p = 0.97 | 1.15 (0.5, 2.33) | 8.7 (8, 9.5) |
|  | 18 hours | 2459 | 1.04 (0.98, 1.09) p = 0.193 | 1.46 (0.66, 2.94) | 8.9 (8, 9.7) |
|  | 24 hours | 2375 | 1.05 (1.01, 1.1) p = 0.022 | 1.78 (0.78, 3.41) | 8.7 (8.1, 9.4) |
| Patients without moderate anemia | 6 hours | 5663 | 0.91 (0.84, 0.97) p = 0.005 | 0.78 (0.38, 1.49) | 10.9 (9.7, 12.2) |
|  | 12 hours | 5660 | 0.94 (0.9, 0.98) p = 0.007 | 1.19 (0.5, 2.33) | 10.9 (9.9, 12) |
|  | 18 hours | 5644 | 0.97 (0.94, 1.01) p = 0.108 | 1.53 (0.68, 3.03) | 10.6 (9.7, 11.8) |
|  | 24 hours | 5508 | 0.99 (0.96, 1.02) p = 0.405 | 1.84 (0.8, 3.52) | 10.6 (9.6, 11.8) |

For moderate anemia patients, increasing fluid balance was associated with increased risk of 28-day mortality at 24 hours after ICU admission (OR 1.05, 95% CI 1.01 - 1.1, p = 0.022) with median fluid balance of 1.78 L (IQR 0.78L - 3.41L). ). Median hemoglobin level overall showed a slight decrease from 8.8g/dL (IQR 7.9g/dL - 9.6g/dL) at 6 hours after ICU admission, to 8.7g/dL at 24 hours after ICU admission (median IQR 8.1g/dL - 9.4g/dL). For patients without moderate anemia, increasing fluid balance was associated with decreased risk of 28-day mortality at 6 hours (OR 0.91, 95% CI 0.84 - 0.97, p=0.005) and 12 hours (OR 0.94, 95% CI 0.9 - 0.98, p=0.007) after ICU admission (Fig 2b, Table 2). Median fluid balance was 0.78L (IQR 0.38L - 1.49L) at 6 hours and 1.19L (IQR 0.5L - 2.33L) at 12 hours after ICU admission. Median hemoglobin level showed a decrease from 10.9g/dL (IQR 9.7g/dL – 12.2g/dL) at 6 hours after ICU admission, to 10.6g/dL (IQR 9.6g/dL – 11.8g/dL) at 24 hours after ICU admission.

We have also newly performed a sensitivity analysis to examine effects of increasing fluid balance on risk 28-day mortality with modified definitions of moderate anemia as 7-9 g/dL and 7-8 g/dL. We have also noted a previous study that found that different early hemoglobin levels may be a predictor of long-term mortality for sepsis patients(7). The results of this sensitivity analysis can be found in ***S9 Fig.*** Our findings indicate that patients without moderate anemia may benefit from more positive fluid balance in the first 24 hours of ICU stay, while no beneficial effect was found in patients with moderate anemia.

**S9a. Visualization of sensitivity analyses results for moderate anemia defined as 7-9g/dL**

**S9a.** Logistic regression results for sepsis patients, where moderate anemia was defined as admission Hb 7-9 g/dL. The ORs represent the risk of 28-day mortality at different observation windows after ICU admission. (a) and (b) show the regression results with and without moderate anemia, respectively. Moderate anemia patients had no significant difference in 28-day mortality (OR 1.06, 95% CI 0.99 – 1.13, p=0.07 at 24 hours) with increasing fluid balance. Conversely, patients without moderate anemia had decreased risk of 28-day mortality (OR 0.9, 95% CI 0.85 – 0.96, p = 0.01 at 6 hours; OR 0.95, 95% CI 0.91 – 0.99, p = 0.008 at 12 hours) with increasing fluid balance. Abbreviations: FB = Fluid balance; ICU = Intensive Care Unit; OR = Odds Ratio

**S9c. Visualization of sensitivity analyses results for moderate anemia defined as 7-8g/dL**

**S9c.** Logistic regression results for sepsis patients, where moderate anemia was defined as admission Hb 7-8g/dL. The ORs represent the risk of 28-day mortality at different observation windows after ICU admission. (a) and (b) show the regression results with and without moderate anemia, respectively. Moderate anemia patients had no significant difference in mortality (OR 1.07, 95% CI 0.96 – 1.2, p = 0.211 at 24 hours) with increasing fluid balance. In contrast, patients without moderate anemia had decreased risk of 28-day mortality (OR 0.92, 95% CI 0.87 – 0.98, p = 0.006 at 6 hours; OR 0.95, 95% CI 0.92 – 0.99, p = 0.011 at 12 hours) with increasing fluid balance. Abbreviations: FB = Fluid balance; ICU = Intensive Care Unit; OR = Odds Ratio

We have added these analyses as well as reference to our methods and results sections of the manuscript.

***1ii. Early fluid resuscitation in different ICU groups must be adjusted according to patients’ hemodynamic parameters.***

***3. ICU patients’ outcome can influence by many different parameters. It is difficult to exclude the mixing factors using logistical regression, and the results lack credibility. In this paper, to confirm the connection between positive fluid balance and hemoglobin levels in mortality, all the confound factors need to be strictly matched. At the same time, the statistical analysis needs to be further improved.***

Response

In order to further adjust for confounding factors as suggested, we have conducted an analysis with propensity score matching. We took fluid balance as a dichotomous (positive or negative) variable, and the probability of having a positive or negative fluid balance was estimated with baseline covariates. The matched cohort had 6,190 patients. As shown below, the result is consistent with our primary findings: patients without moderate anemia had decreased risk 28-day mortality with increasing fluid balance (OR 0.94, 95% CI 0.89 - 0.99 at 12 hours, p = 0.026). In moderate anemia patients, there was an increased risk of 28-day mortality (OR 1.08, 95% CI 1.02 - 1.14at 12 hours, p = 0.006) with increasing fluid balance. Detailed results of this can be seen in the newly added ***S8 Fig and Table*** (***Fig S8b*** is shown below).

**S8b. Visualization of regression results after propensity score matching**

**S8b.** Logistic regression results for cohort after propensity score matching. The ORs represent the risk of 28-day mortality, with positive fluid balance at different observation windows after ICU admission. (a) and (b) show the regression results for patients with and without moderate anemia, respectively. Patients without moderate anemia had decreased risk of 28-day mortality with increasing fluid balance (OR 0.94, 95% CI 0.89 - 0.99 at 12 hours, p = 0.026). In moderate anemia patients, there was an increased risk of 28-day mortality (OR 1.08, 95% CI 1.02 - 1.14at 12 hours, p = 0.006) with increasing fluid balance. Abbreviations: FB = Fluid balance; ICU = Intensive Care Unit; OR = Odds Ratio

We do hope that the above responses have adequately addressed your reviews.

Thank you very much once again for your consideration.

Yours Sincerely,

Dr Sandra M. Y. Tan, MBBS

Division of Advanced Internal Medicine, Department of Medicine, National University Hospital

1E Lower Kent Ridge Road

National University Health System Level 10

Singapore 119228

sandra_tan@nuhs.edu.sg

Dr Kay Choong See, MBBS, MPH, FCCP

Division of Respiratory and Critical Care Medicine, Department of Medicine, National University Hospital

1E Lower Kent Ridge Road

National University Health System Level 10

Singapore 119228

kay_choong_see@nuhs.edu.sg

**References**

1.	Maitland K, Kiguli S, Opoka RO, Engoru C, Olupot-Olupot P, Akech SO, et al. Mortality after fluid bolus in African children with severe infection. N Engl J Med. 2011;364(26):2483-95.

2.	Andrews B, Semler MW, Muchemwa L, Kelly P, Lakhi S, Heimburger DC, et al. Effect of an Early Resuscitation Protocol on In-hospital Mortality Among Adults With Sepsis and Hypotension: A Randomized Clinical Trial. JAMA. 2017;318(13):1233-40.

3.	Maitland K, George EC, Evans JA, Kiguli S, Olupot-Olupot P, Akech SO, et al. Exploring mechanisms of excess mortality with early fluid resuscitation: insights from the FEAST trial. BMC Med. 2013;11:68.

4.	Maiden MJ, Finnis ME, Peake S, McRae S, Delaney A, Bailey M, et al. Haemoglobin concentration and volume of intravenous fluids in septic shock in the ARISE trial. Crit Care. 2018;22(1):118.

5.	Rivers E, Nguyen B, Havstad S, Ressler J, Muzzin A, Knoblich B, et al. Early goal-directed therapy in the treatment of severe sepsis and septic shock. N Engl J Med. 2001;345(19):1368-77.

6.	Pro CI, Yealy DM, Kellum JA, Huang DT, Barnato AE, Weissfeld LA, et al. A randomized trial of protocol-based care for early septic shock. N Engl J Med. 2014;370(18):1683-93.

7.	Qi D, Peng M. Early Hemoglobin Status as a Predictor of Long-Term Mortality for Sepsis Patients in Intensive Care Units. Shock. 2021;55(2):215-23.
